# Targeting NINJ1-Mediated Plasma Membrane Rupture in Tubular Epithelial Cell Prevents Inflammatory Response in Acute Kidney Injury

**DOI:** 10.7150/ijbs.115364

**Published:** 2025-08-11

**Authors:** Siyan Zhou, Qigang Lan, Wang Xin, Yaqin Wang, Aihong Zhang, Jiachuan Xiong, Liangjing Lv, Yan Li, Ling Nie, Shaozong Qin, Jinrun Zhou, Shuiqin Gong, Shaobo Wang, Yinghui Huang, Jinghong Zhao

**Affiliations:** Department of Nephrology, Chongqing Key Laboratory of Prevention and Treatment of Kidney Disease, Chongqing Clinical Research Center of Kidney and Urology Diseases, Xinqiao Hospital, Army Medical University (Third Military Medical University), Chongqing, 400037, China.

**Keywords:** nerve injury-induced protein 1 (NINJ1), plasma membrane rupture, damage-associated molecular pattern, inflammation, acute kidney injury.

## Abstract

Damage-associated molecular patterns (DAMPs)-induced sterile inflammation is considered as a typical feature of acute kidney injury (AKI). Plasma membrane rupture in renal tubular epithelial cells (RTECs) is the major cause of DAMP release and nerve injury-induced protein 1 (NINJ1) has recently emerged as an executor of plasma membrane rupture, while its role in AKI pathophysiology remains largely unknown. Here, we show upregulated NINJ1 expression and oligomerization in renal tubules among human biopsies and mouse models as well as in cultured RTECs after AKI, accompanied by plasma membrane rupture, increased DAMP release and inflammatory response. Furthermore, knockdown of NINJ1 or inhibition of its oligomerization effectively prevents plasma membrane rupture in RTECs, thereby alleviating DAMP-induced inflammatory response and renal tubular injury. Mechanistically, the ETS transcription factor (ELK1) is identified as a novel transcription factor for NINJ1 during AKI, especially ELK1 phosphorylation at Ser^383^ significantly enhances its transcriptional activity. Importantly, genetic silencing of NINJ1 or pharmacological inhibition of Ser^383^-phosphorylated ELK1 can protect against AKI and improve AKI prognosis. Collectively, these findings highlight the ELK1-NINJ1 axis as a pivotal regulator of plasma membrane rupture in RTECs upon AKI, suggesting that it may serve as a potential target for AKI treatment and prognosis improvement.

## Introduction

Acute kidney injury (AKI) is a severe and urgent health issue with high morbidity and mortality 1, 2. The disease is characterized by a rapid and sharp decline in renal function 3. AKI with poor prognosis is associated with significant long-term sequelae such as progressive chronic kidney disease (CKD) and increasing mortality 4. Unfortunately, no effective pharmacologic strategies for the prevention and treatment of AKI have been reported to date 5, and it is important to improve our understanding of AKI and identify novel therapeutic opportunities.

Renal tubular epithelial cell (RTEC) injury and death are the primary pathological characteristics of AKI 6. Several distinct RTEC death events have been reported after AKI, including pyroptosis, necroptosis and ferroptosis, which ultimately lead to plasma membrane rupture and cell lysis 7-9. Of note, damage-associated molecular patterns (DAMPs) released upon plasma membrane rupture or cell death can interact with the corresponding pattern recognition receptors on RTECs themselves, resulting in activation of the inflammatory response 10, 11. Furthermore, the release of DAMPs into the extracellular space can also recruit and activate immune cells, such as macrophages and neutrophils, thereby exacerbating renal injury 12. Given DAMPs provoke a rapid course of sterile inflammation and exacerbate subsequent tissue injury 13, preventing the release of DAMPs is currently considered a novel therapeutic strategy to improve AKI outcomes.

Nerve injury-induced protein 1 (Ninjurin1, NINJ1) is a 16-kDa cell-surface protein that adopts a three-helix conformation and is located in the plasma membrane 14. NINJ1 is widely expressed in a variety of cells, including neurons, myeloid cells and cancer cells 15-17. Notably, current evidence identifies NINJ1 as a common executor of plasma membrane rupture in pyroptosis, necrosis, and post-apoptotic lysis 18. When NINJ1 is activated, its extracellular domain oligomerizes and subsequently caps membrane edges, disrupts membranes, thus non-selectively releasing DAMPs from dying cells 19, 20. Interestingly, accumulating studies have suggested that NINJ1 plays an intrinsic role in various inflammatory processes by regulating leukocyte infiltration 21, 22, indicating that NINJ1 may regulate sterile inflammation, while the expression pattern and the role of NINJ1 in AKI remain unclear. Since AKI is also closely associated with sterile inflammation 23, the role of NINJ1 in regulating sterile inflammation and modulating tubular injury during AKI deserves further study.

In this study, using human biopsy specimens and multiple mouse models of AKI, we reveal the expression pattern of NINJ1 in RTECs, and identify its obvious correlation with renal inflammation and injury. Furthermore, we show that upon AKI, both *in vivo* and *in vitro*, plasma membrane NINJ1 expression is elevated and rapidly oligomerized to induce plasma membrane rupture, thereby releasing a large number of DAMPs into the extracellular space to aggravate pro-inflammation cytokine production. Molecularly, we identify ETS transcription factor (ELK1) as a novel regulator of NINJ1 transcriptional expression through direct binding to the NINJ1 promoter region. Importantly, targeting ELK1 phosphorylation and NINJ1 may protect against AKI and improve AKI prognosis.

## Material and Methods

Further details regarding the all methods are provided in the Supplementary Methods.

### Human renal biopsy specimens

Human renal specimens were obtained from patients undergone kidney biopsy at the Department of Nephrology of Xinqiao Hospital. 23 patients with acute tubular necrosis (ATN) and 20 patients with no significant abnormal changes were enrolled in this study. The detailed information for these enrolled patients can be found in Supplementary Table 1. Informed consent was obtained from all patients. Renal biopsies from these patients were used for NINJ1 immunohistochemical staining.

### Animals

Male C57BL/6J mice (8 weeks old) were purchased from Chongqing Tengxin Bioscience (Chongqing, China). The ischemia-reperfusion injury (IRI)-AKI model was established as previously described 24. In brief, after general anesthesia, bilateral renal vessels of the mice were clamped for 30 minutes at 37 ℃. For sham operation, the same procedure without arterial clamps was performed. To construct IRI induced AKI to CKD model, bilateral renal ischemia of 35 minutes, followed by reperfusion for 2 or 4 weeks was employed. For cisplatin (Cis)-AKI model, 25 mg/kg of cisplatin or vehicle (saline) was intraperitoneally injected into mice for 3 days as described previously 25. For folic acid (FA)-AKI model, 250 mg/kg FA or vehicle (0.3 M sodium bicarbonate) was intraperitoneally injected into mice and were euthanized 2 days later as described previously 25.

### Cell culture

HK-2 (human kidney 2) cells, a human proximal tubular epithelial cell line, were purchased from the American Tissue Culture Collection (ATCC, Manassas, VA, USA) and were cultured in DMEM/F12 medium containing 10% fetal bovine serum (FBS) (Mediatech Inc., Herndon, VA, USA) at 37 ℃ in a humidified atmosphere with 5% CO_2_. Human monocyte cell line (THP-1) was cultured in RPMI 1640 medium supplemented with 10% FBS (Mediatech Inc). For differentiation, THP-1 monocytes were treated with phorbol 12-myristate 13-acetate (PMA, 100 ng/mL) for 24 hours to induce macrophage phenotype. For hypoxia/reoxygenation (H/R) model, HK-2 cells were exposed to 24 hours of hypoxia condition (94%N_2_, 1%O_2_, 5%CO_2_, glucose and serum-free DMEM/F12 medium) followed by 1 h, 3 h, 6 h, 12 h, and 24 h of reoxygenation (95% air and 5% CO_2_, DMEM/F12 medium with 10% FBS).

### Transfection of siRNA

According to the manufacturer's protocol, HK-2 cells were transfected with siRNA (Ninj1, ELK1, interferon regulatory factor 1 (IRF1), and Yin-Yang 1 (YY1)) by using Lipofectamine 3000 (L3000075, Invitrogen, Carlsbad, CA, USA) in Opti-MEM (Hyclone, Logan, Utah, USA). Successful transfection was confirmed by quantitative PCR (qPCR) and western blot analysis. The sequences of siRNAs are provided in Supplementary Table 2.

### Adeno-Associated Virus-Mediated Ninj1 Knockdown *in vivo*

For knockdown of Ninj1 *in vivo*, shRNA targeting mouse Ninj1 was cloned and packaged into an adeno-associated virus serotype 9 (AAV9) vector bearing the GFP linked Ksp-cadherin promoter (a unique, tissue-specific member of the cadherin family that is exclusively expressed in RTECs). Ninj1 shRNA sequence was as follows: 5'- CGTGGTCAACATCTTCATTACTAGTGAAGCCACAGATGTAGTAATGAAGATGTTGACCACGA-3'. The shScramble was prepared as a negative control sequence. After sequencing ensured accuracy of the vector, adeno-associated virus was packaged, purified and titrated by Vigene Biosciences (Shandong, China). The titers of adeno-associated virus serotype 9 (AAV9)-short hairpin Ninj1 plasmid (AAV9-shNinj1) and AAV9-vector were approximately 9.64×10^13^ viral genomes/ml. AAV9-shNinj1 or AAV9-vector was delivered via renal pelvis injection into C57BL/6J mice at 1×10^12^ copies 26. For renal pelvis injection, mice were anesthetized and the kidney was exposed through the flank incision. The renal pelvis was exposed, and AAV9 particles were injected into the renal pelvis using a 31-gauge needle. At 4 weeks after injection, immunofluorescence and western blot analysis were performed to confirm that Ninj1 was knockdown in the mouse kidneys.

### Native-PAGE

Native-PAGE was conducted as described previously 18. HK-2 cells and kidney tissues were lysed with native-PAGE lysis buffer (150 mM NaCl, 1% Digitonin, 50 mM Tris pH7.5, and 1× Complete Protease Inhibitor). After centrifuging at 20800g for 30 min, protein concentration of lysates was measured using BCA kit (P00009, Beyotime Biotechnology, Shanghai, China) to ensure equal loading of proteins. Subsequently, lysates were mixed with 4× NativePAGE sample buffer and subjected to Native-PAGE. Detailed information of antibodies is listed in Supplementary Table 3.

### Silver staining

For visualization of secreted proteins in culture supernatant, HK-2 cells were rinsed with phosphate buffer saline (PBS) three times and cultured in no-FBS DMEM/F12 medium for 6 h after H/R treatment. Culture supernatant was collected as described in previous study 27. Then, 10 μl of the supernatant was run on SDS-PAGE and proteins were silver stained by using Fast Silver Stain Kit (Beyotime Biotechnology, Shanghai, China).

### Cell assays and cytokine measurements

Lactate dehydrogenase (LDH) content in culture supernatants and mouse serum was determined using a LDH colorimetric assay kit (Promega, Madison, USA) following the manufacturer's instructions. Interleukin 1β (IL-1β) and high mobility group box 1 (HMGB1) content in culture supernatants and mouse serum were determined respectively using IL-1β ELISA Kit and HMGB1 ELISA Kit (Jianglai Biological, Shanghai, China).

### Scanning electron microscope (SEM)

Cells were fixed with 2.5% glutaraldehyde overnight, and then washed with PBS three times. Sample was dehydrated through a graded series of ethanol (30, 50, 70, 95 and 100%) and dried by tertiary butanol method. The coverslip was sputtered with gold before observation at 15 kV under a Hitachi S-3400N SEM.

### Reverse transcription and qPCR

Total RNA was extracted from the mouse kidneys and HK-2 cells using RNAeasy™ Plus Animal RNA Isolation Kit with Spin Column (R0032, Beyotime Biotechnology). The RNA was reverse transcribed into cDNA using RT Master Mix for qPCR kit (HY-K0511, MedChemExpress, New Jersey, USA). The mRNA expression was detected by qPCR using SYBR Green qPCR Master Mix (HYK0523, MedChemExpress) on a CFX96 Real-Time system. The sequences of primers for qPCR are listed in Supplementary Table 4 and Supplementary Table 5. The mRNA expression levels of the target genes were normalized relative to β-actin and calculated by the 2^-ΔΔCT^ method.

### Statistics analysis

Statistical analysis was performed using GraphPad Prism 8. All experimental data were shown as the mean ± standard deviation (SD), each experiment represent a minimum of three independent samples. Comparisons were analyzed using a two-tailed, paired Student's *t*-test, one-way analysis of variance (ANOVA) or Pearson's correlation test. *P* < 0.05 were considered statistically significant.

## Results

### NINJ1 expression and oligomerization is highly induced in human biopsies and mouse models after AKI

**Figure 1:**
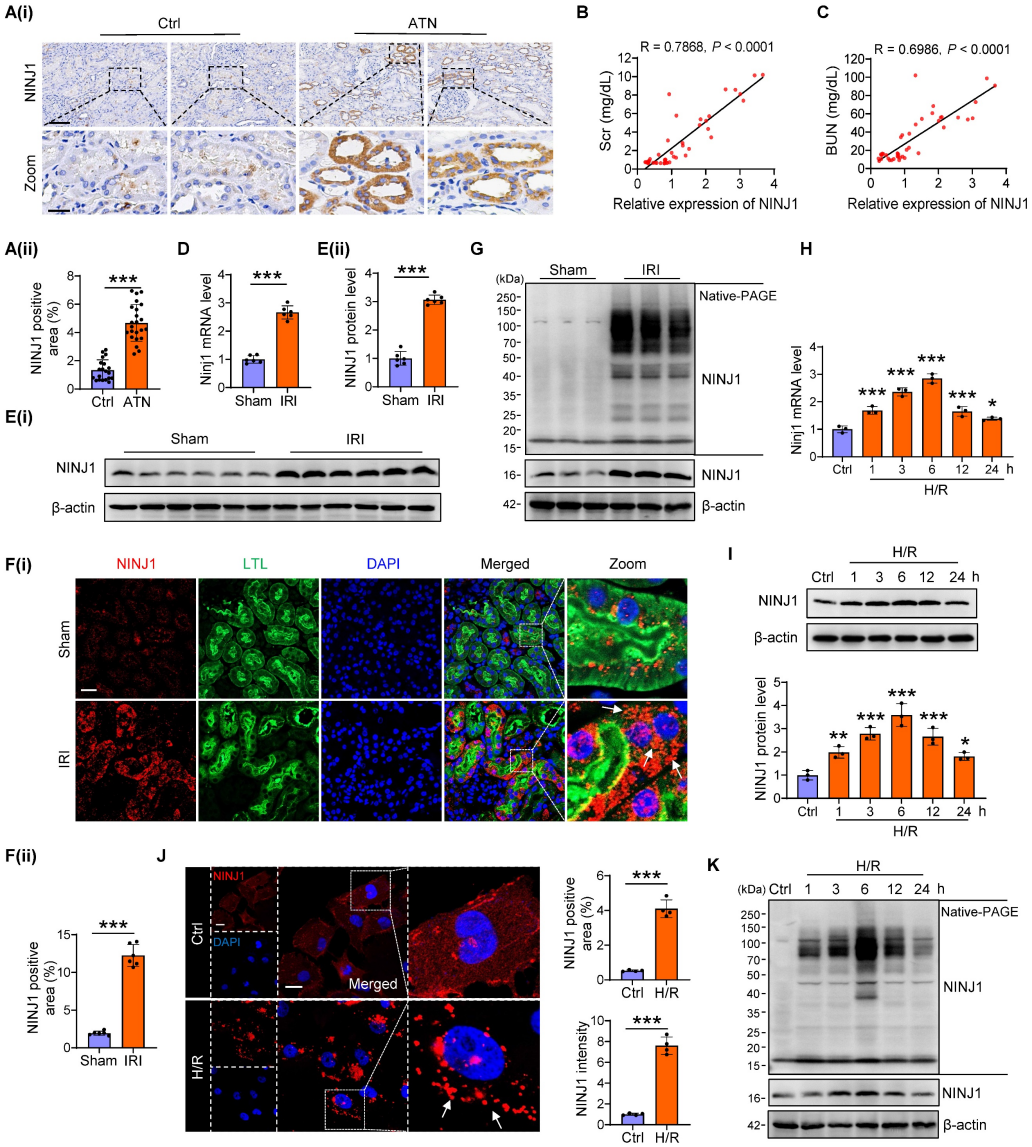
** NINJ1 expression and oligomerization is highly induced in human biopsies and mouse models after AKI**. **(A)** Representative immunohistochemical (IHC) imaging of NINJ1 in the kidney biopsies from acute tubular necrosis (ATN) patients (n = 23) or control (n = 20), and quantification of IHC images by using ImageJ software. Scale bar = 100 μm (top); 25μm (down). **(B, C)** The correlation analysis between NINJ1 expression and serum creatinine (Scr) (B) or blood urea nitrogen (BUN) (C). **(D-F)** Relative expression of NINJ1 in renal tissues of sham and ischemia reperfusion injury (IRI)-induced acute kidney injury (AKI) mice, determined respectively by qPCR (D) (n = 6), western blot (E) (n = 6), and immunofluorescence (F) (n = 6). Scale bar = 50 μm. LTL, Lotus tetragonolobus lectin. DAPI, 40 ,6- diamidino-2-phenylindole. White arrows indicate oligomerized NINJ1. **(G)** Native-PAGE analysis of endogenous NINJ1 in renal tissues of sham and IRI-induced AKI mice. **(H-J)** Relative expression of NINJ1 in human kidney 2 (HK-2) cells with or without hypoxia-reoxygenation (H/R) treatment, determined respectively by qPCR (H) (n = 3), western blot (I) (n = 3), and immunofluorescence (J) (n = 4). Scale bar = 50 μm. White arrows indicate oligomerized NINJ1. **(K)** Native-PAGE analysis of endogenous NINJ1 in HK-2 cells with or without H/R treatment. Data are shown as mean ± standard deviation (SD). *P < 0.05; **P < 0.01; ***P < 0.001.

Initially, we confirmed the expression pattern of NINJ1 in renal biopsy specimens from 23 patients with ATN and 20 patients without detectable ATN (Supplementary Table 1). Immunochemistry showed an upregulated expression of NINJ1 in ATN (Figure 1A), which was positively correlated with the serum creatinine (Scr) and blood urea nitrogen (BUN) levels (Figure 1B, C), hinting that increased NINJ1 expression may associated with AKI pathogenesis. To further clarify the role of NINJ1 in AKI, we established three AKI mouse models, including IRI-induced AKI, Cis-induced AKI, and FA-induced AKI (Supplementary Figure 1A-I), and detected a significant increase in NINJ1 expression at both mRNA and protein levels after AKI (Figure 1D, E and Supplementary Figure 1J-N). Interestingly, NINJ1 was significantly upregulated in proximal tubules after AKI and characterized as discrete plasma membrane clusters or assemblies, suggesting that NINJ1 undergoes oligomerization (Figure 1F). Accordingly, we observed that endogenous NINJ1 was shifted to a high molecular weight aggregate after IRI by native PAGE (Figure 1G). Given that NINJ1 oligomerization mediates plasma membrane rupture, we detected that the LDH, a well-known indicator of plasma membrane rupture, as well as HMGB1 and IL-1β, the common proinflammatory DAMPs 18, were released at high levels in the serum of AKI mice (Supplementary Figure 1O). DAMPs present in the AKI milieu may interact with pattern recognition receptors on RTECs to further exacerbate the inflammation 28. Consistently, the proinflammatory factors, such as tumor necrosis factor alpha (TNF-α), interleukin 6 (IL-6) and, monocyte chemotactic protein 1 (MCP-1) were significantly upregulated in RTECs of AKI mice (Supplementary Figure 1P). *In vitro*, significantly increased expression and oligomerization of NINJ1 were also observed in HK-2 cells at 6 hours after H/R treatment (Figure 1H-K), accompanied by dramatic upregulation of LDH, HMGB1 and IL-1β in the cell supernatant and proinflammatory factors in HK-2 cells (Supplementary Figure 1Q, R). Collectively, these results indicate that the NINJ1 expression and oligomerization are highly induced in RTECs during AKI, together with plasma membrane rupture, DAMP release and inflammatory response.

### Inhibition of NINJ1 oligomerization attenuates DAMP release and inflammatory response in RTECs

**Figure 2:**
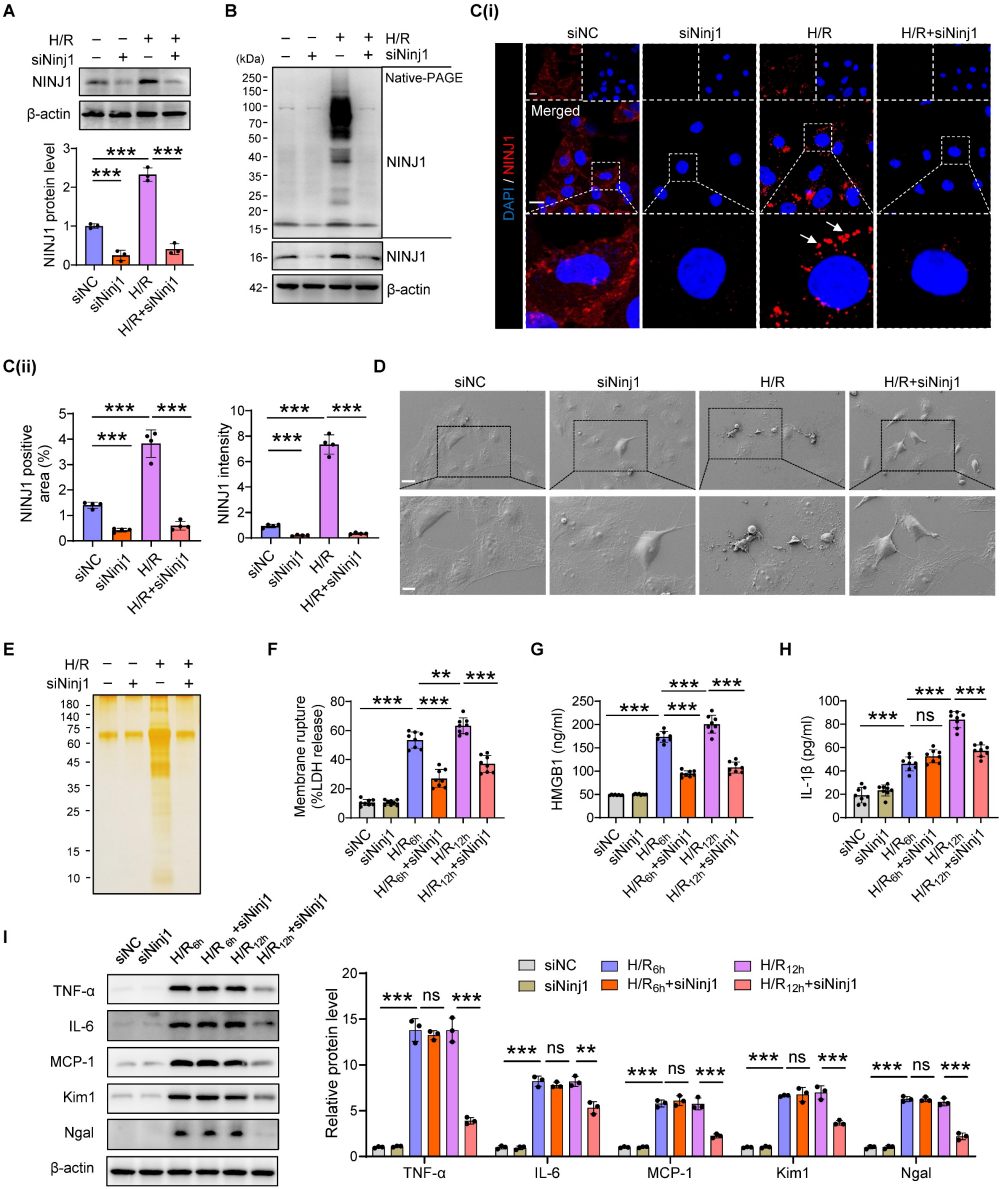
** Ninj1-deficiency blocks DAMP release and inflammatory response under H/R condition.** HK-2 cells were transfected with siRNA targeting Ninj1 (siNinj1) or non-targeted control (siNC) under normoxia or H/R conditions.** (A)** Western blot analysis of NINJ1 expression following the described treatment (n = 3). **(B)** Native-PAGE analysis of endogenous NINJ1 in HK-2 cells following the described treatment. **(C)** Representative immunofluorescence staining of NINJ1 in HK-2 cells following the described treatment. Quantification of the positive area and mean fluorescence intensity of NINJ1 (n = 4). Scale bar = 50 μm. White arrows indicate oligomerized NINJ1. **(D)** Scanning electron microscope (SEM) observation of HK-2 cells following the described treatment. Scale bar = 20 μm (top); 10 μm (down).** (E)** Silver staining of released proteins in culture supernatant of HK-2 cells following the described treatment.** (F-H)** Release of lactate dehydrogenase (LDH, F), high mobility group box 1 (HMGB1, G) and interleukin-1β (IL-1β, H) in culture supernatant of HK-2 cells after reoxygenation at indicate time (n = 8). **(I)** Western blot analysis of tumor necrosis factor alpha (TNF-α), interleukin 6 (IL-6), monocyte chemotactic protein 1 (MCP-1), kidney injury molecule 1 (*Kim1*), and neutrophil gelatinase-associated lipocalin (*Ngal*) in HK-2 cells at indicated time (n = 3). Data are shown as mean ± SD. **P < 0.01; ***P < 0.001. ns: no significance.

**Figure 3:**
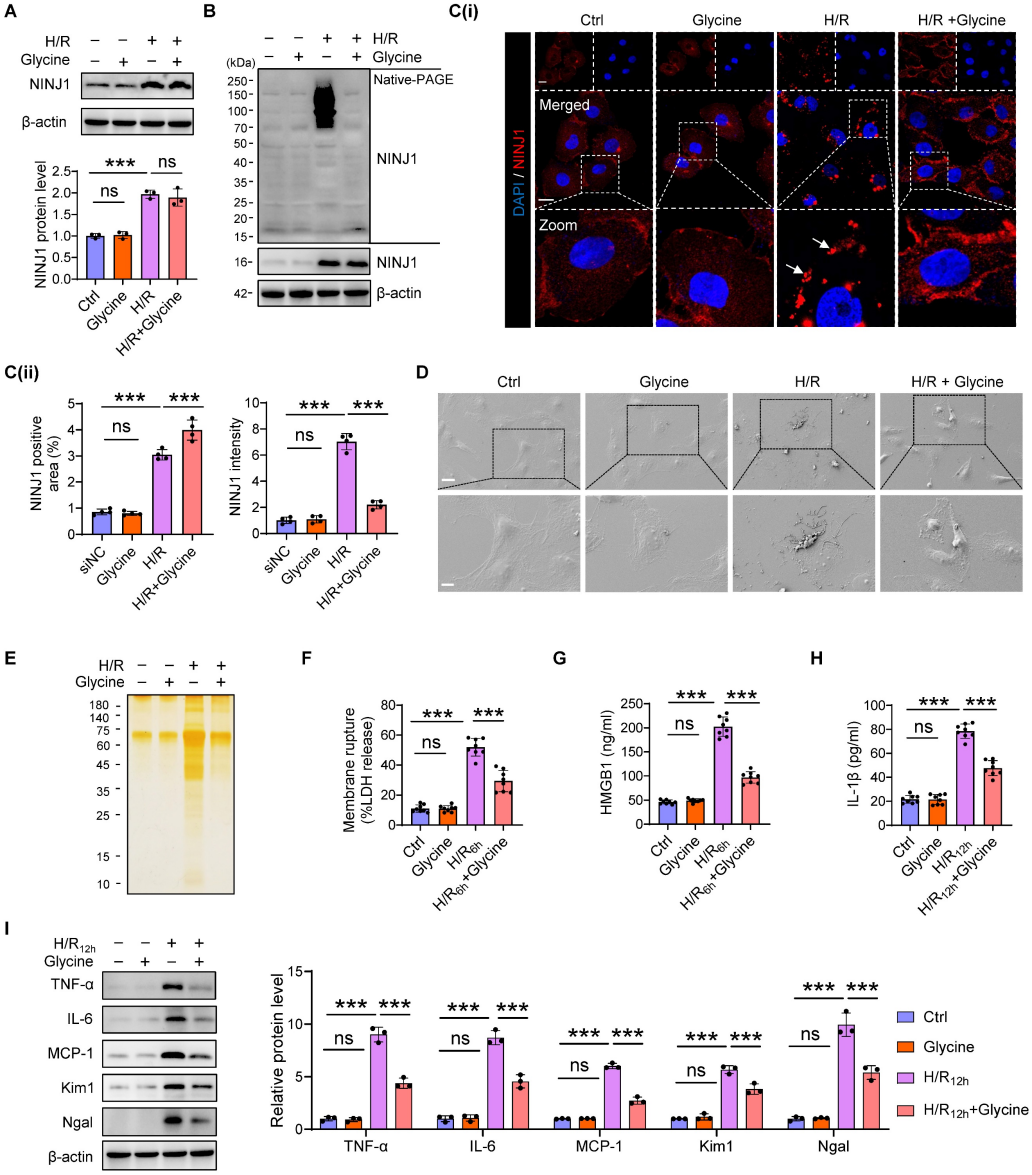
** Inhibition of NINJ1 oligomerization prevents plasma membrane rupture.** HK-2 cells were treated with or without glycine (5mM) under normoxia or H/R conditions. **(A)** Western blot analysis of NINJ1 expression following the described treatment (n = 3). **(B)** Native-PAGE analysis of endogenous NINJ1 in HK-2 cells following the described treatment. **(C)** Representative immunofluorescence staining of NINJ1 in HK-2 cells following the described treatment. Quantification of the positive area and mean fluorescence intensity of NINJ1 (n = 4). Scale bar = 50 μm. White arrows indicate oligomerized NINJ1. **(D)** SEM observation of HK-2 cells following the described treatment. Scale bar = 20 μm (top); 10 μm (down).** (E)** Silver staining of released proteins in culture supernatant of HK-2 cells following the described treatment.** (F-H)** Release of LDH (F) and HMGB1 (G) in culture supernatant of HK-2 cells after 6 hours of reoxygenation and IL-1β (H) at 12 hours after reoxygenation (n = 8).** (I)** Western blot analysis of TNF-α, IL-6, MCP-1, *Kim1*, and *Ngal* in HK-2 cells following by 12 hours of reoxygenation (n = 3). Data are shown as mean ± SD. ***P < 0.001; ns: no significance.

To verify the detailed function of NINJ1 and its oligomerization in RTECs in response to AKI, HK-2 cells were transfected with siRNA against NINJ1 (siNINJ1) *in vitro* (Figure 2A and Supplementary Figure 2A, B). As shown, the absence of NINJ1 prevented its oligomerization (Figure 2B, C). Moreover, H/R induced pyroptosis-like features in RTECs, characterized by the development of large bubbles in the plasma membrane and the abrupt disintegration of cadavers, while NINJ1 deletion significantly attenuates bubble disintegration (Figure 2D). Notably, silver staining revealed that plasma membrane rupture released more proteins into the supernatant after H/R treatment, while NINJ1 deletion dramatically diminished the proteins released (Figure 2E), suggesting that NINJ1 knockdown could attenuate DAMPs released. Meanwhile, we found that NINJ1 deletion significantly reduced LDH and HMGB1 release at 6 hours after H/R, but IL-1β level was comparable (Figure 2F-H), which may be attributed to the fact that NINJ1 did not affect IL-1β release 18. Interestingly, we detected a decrease in IL-1β release at 12 hours after H/R with NINJ1 knockdown (Figure 2H), in parallel with downregulated TNF-α, IL-6 and MCP-1 levels at 12 hours but not 6 hours after H/R (Figure 2I and Supplementary Figure 2C-E). Correspondingly, knockdown of NINJ1 also attenuated HK-2 cells injury, as evidenced by lower expression of kidney injury molecule 1 (*Kim1*) and neutrophil gelatinase-associated lipocalin (*Ngal*) (Figure 2I and Supplementary Figure 2F, G). These findings suggest that knockdown of NINJ1 prevents its oligomerization, thereby attenuating further RTEC injury and inflammatory response. Furthermore, glycine, a cytoprotective agent 29, was treated to HK-2 cells to prevent the oligomerization of NINJ1. Glycine alone treatment did not affect the expression or oligomerization of NINJ1 (Figure 3A-C). However, glycine treatment the significantly inhibited NINJ1 oligomerization under H/R condition (Figure 3A-C), accompanied by the absence of membrane rupture, decreased release of DAMPs and reduced inflammation in HK-2 cells (Figure 3D-I and Supplementary Figure 3A-E). Overall, these lines of evidence further emphasize that NINJ1 contributes to RTEC damage and inflammation by inducing plasma membrane rupture and DAMP release.

### Silencing of NINJ1 protects against AKI and improves AKI prognosis

**Figure 4:**
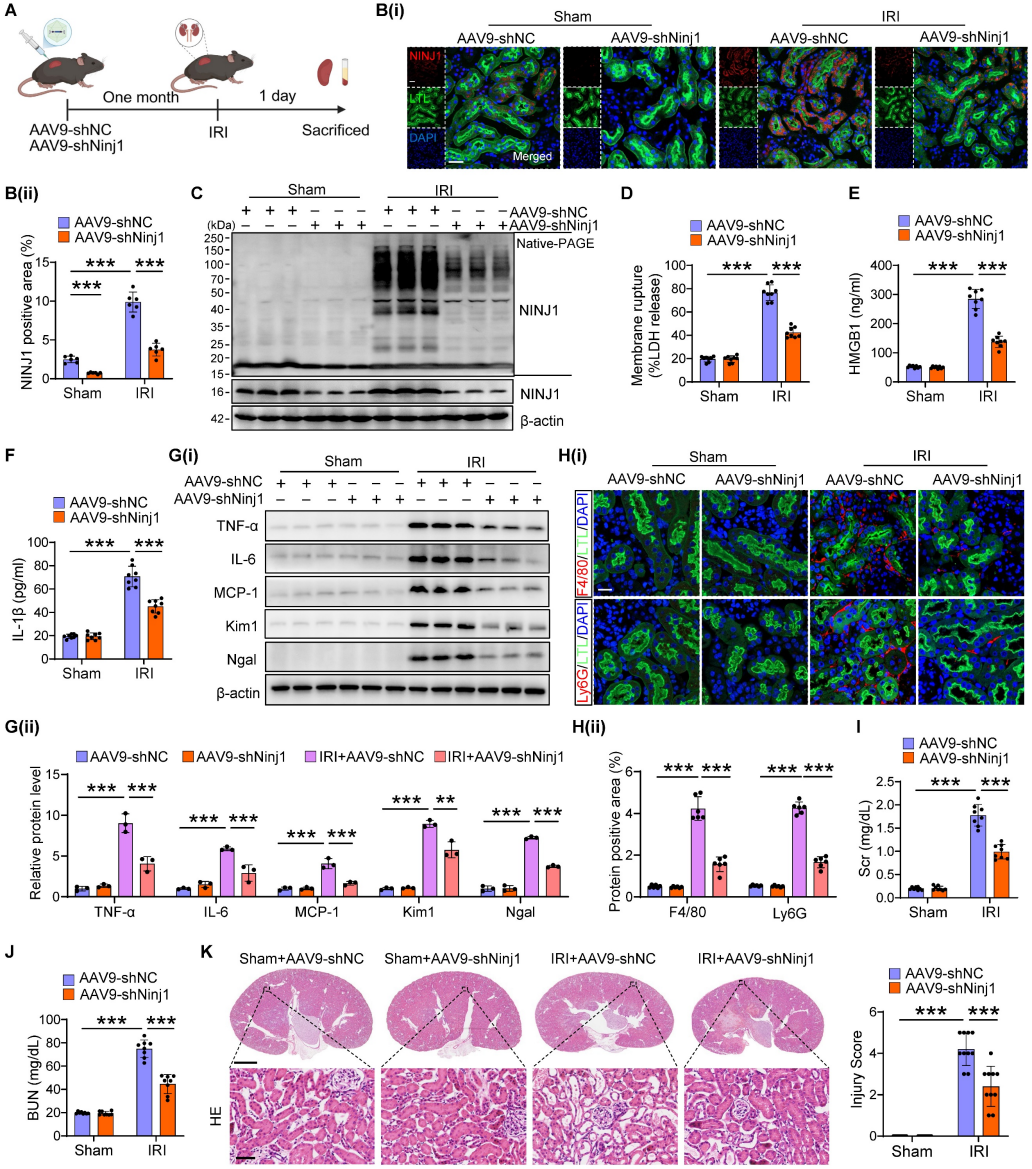
** Renal tubule-specific NINJ1 knockdown alleviates renal injury in IRI-induced AKI mice. (A)** Schematic representation of the adeno-associated virus serotype 9 (AAV9)-short hairpin Ninj1 plasmid (AAV9-shNinj1) administration in C57BL/6J mice for one month before being subjected to IRI.** (B)** Representative immunofluorescence images and quantification of NINJ1 from mice injected with AAV9-shNC or AAV9-shNinj1 with or without IRI. Scale bar = 50 μm.** (C)** Native-PAGE analysis of endogenous NINJ1 in renal tissues from mice following the described treatment. **(D-F)** Release of LDH (D), HMGB1 (E) and IL-1β (F) in renal tissues from mice following the described treatment (n = 8).** (G)** Western blot analysis of TNF-α, IL-6, MCP-1, *Kim1* and *Ngal* expression in renal tissues from mice following the described treatment (n = 3).** (H)** Confocal microscopy showing the expression of F4/80 or Ly6G in renal tissues from mice following the described treatment. Scale bar = 50 μm.** (I, J)** Levels of Scr (I) and BUN (J) in renal tissues from mice following the described treatment (n = 8).** (K)** Hematoxylin and eosin (HE) staining and injury score in renal tissues from mice following the described treatment (n = 8). Scale bars = 1.25 mm (top); 50 μm (down). Data are shown as mean ± SD. ***P < 0.001.

**Figure 5:**
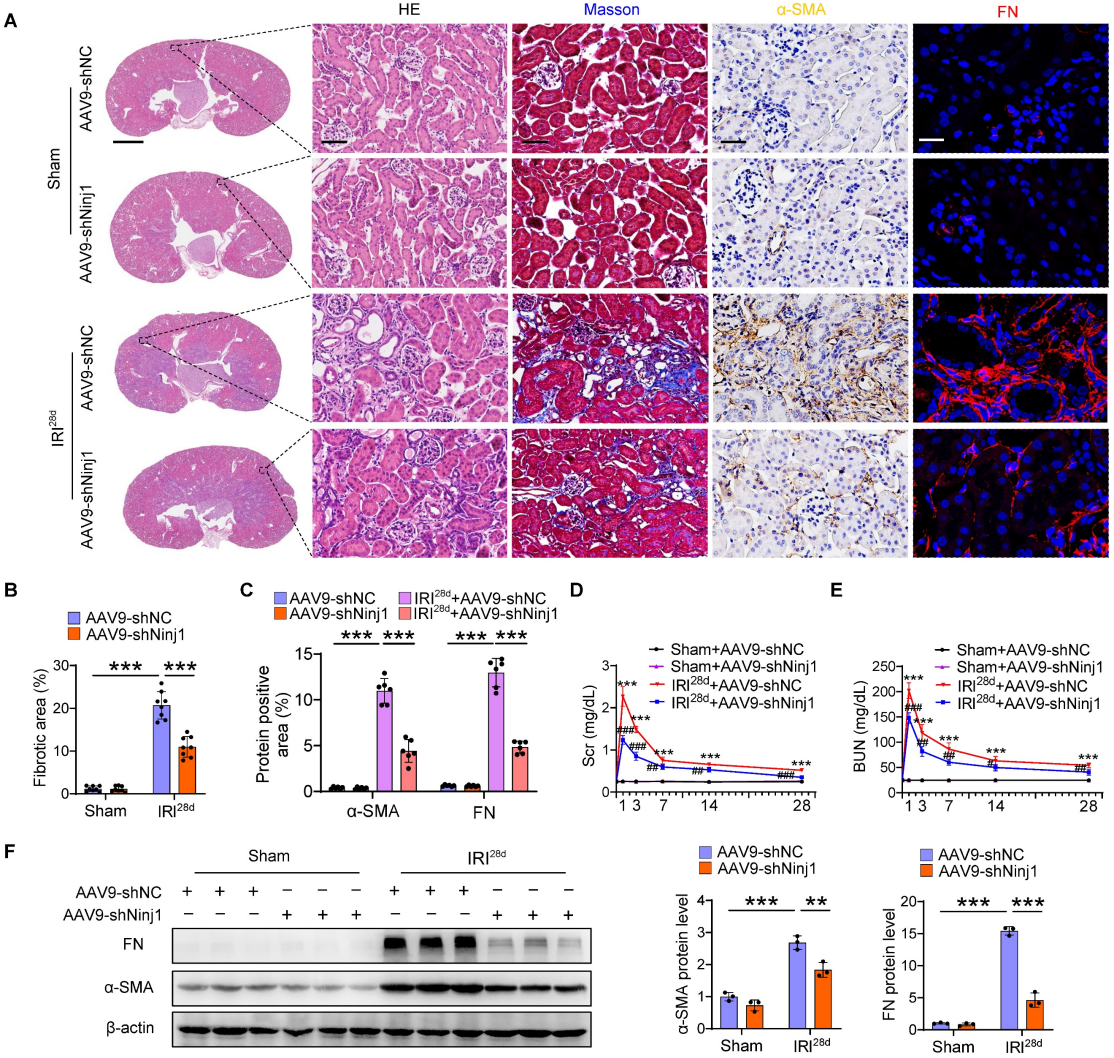
** Renal tubule-specific NINJ1 knockdown attenuates the transition of AKI to CKD.** AAV-shNC and AAV-shNinj1 mice were subjected to sham or IRI, then sacrificed on the 28^th^ day after reperfusion. **(A)** Representative imaging of HE, Masson, alpha smooth muscle actin (a-SMA), and fibronectin (FN) staining in renal tissues of mice. Scale bars = 1.25 mm (far left); 50 μm (others). **(B)** Quantitative analysis of the fibrotic area (n = 8).** (C)** Positive area of a-SMA and FN in kidneys in A (n = 6). **(D, E)** The levels of Scr (D) and BUN (E) over time from mice following the described treatment (n = 8). **(F)** Western blot analysis of the protein levels of FN and α-SMA in renal tissues from mice following the described treatment (n = 3). Data are shown as mean ± SD. ***P < 0.001 versus sham; # *P* < 0.05, ## *P* < 0.01; ### *P* < 0.001 versus IRI.

To determine the therapeutic potential of NINJ1 in AKI mouse models, we silenced NINJ1 in mice using an adeno-associated virus serotype 9 (AAV9)-short hairpin Ninj1 plasmid (AAV9-shNinj1). Accordingly, we observed that AAV9-shNinj1 was successfully transfected in RTECs, and the decreased expression of NINJ1 was validated using western blot and immunofluorescence (Figure 4A, B and Supplementary Figure 4A, B). Compared with mice receiving AAV9-shNC, AAV9-shNinj1 treatment significantly relieved IRI-induced NINJ1 oligomerization (Figure 4C), leading to decreased LDH and DAMPs release (Figure 4D-F), proinflammatory cytokine production (Figure 4G and Supplementary Figure 4C-G). Notably, HMBG1 and IL-1β are not only derived from immune cells, such as macrophages and neutrophils, but can also recruit them to activate the immune response 12. In favor of this knowledge, we observed a significant infiltration of macrophages and neutrophils into the renal interstitium after IRI, while decreased infiltration of these cells in AAV9-shNinj1 mice (Figure 4H). Besides, we established a co-culture system, and found that macrophages co-cultured with H/R-treated HK-2 cells exhibited significantly faster migration (Supplementary Figure 5A and B) and a pro-inflammation phenotype (Supplementary Figure 5C-G), as evidenced by the upregulation of CD86 and inducible nitric oxide synthase (iNOS). Conversely, NINJ1 knockdown effectively prevented both macrophage migration and the pro-inflammatory phenotype transition (Supplementary Figure 5A-G). Correspondingly, we found that the disruption of NINJ1 lowered serum Scr and BUN levels and mitigated tubular injury (Figure 4I-K). Furthermore, we also explored the effect of NINJ1 in AKI-to-CKD transition. Treatment with AAV9-shNinj1 significantly decreased collagen deposition, α smooth muscle actin (α-SMA) and fibronectin (FN) expression at day 28 after AKI, consequently rescued the worsen renal function (Figure 5A-F). Above all, these data demonstrate that disruption of NINJ1 expression in RTECs might represent a promising strategy for protecting against AKI and improving the prognosis of AKI.

### ELK1 transcriptionally upregulates NINJ1 expression by directly binding to NINJ1 promoter

**Figure 6:**
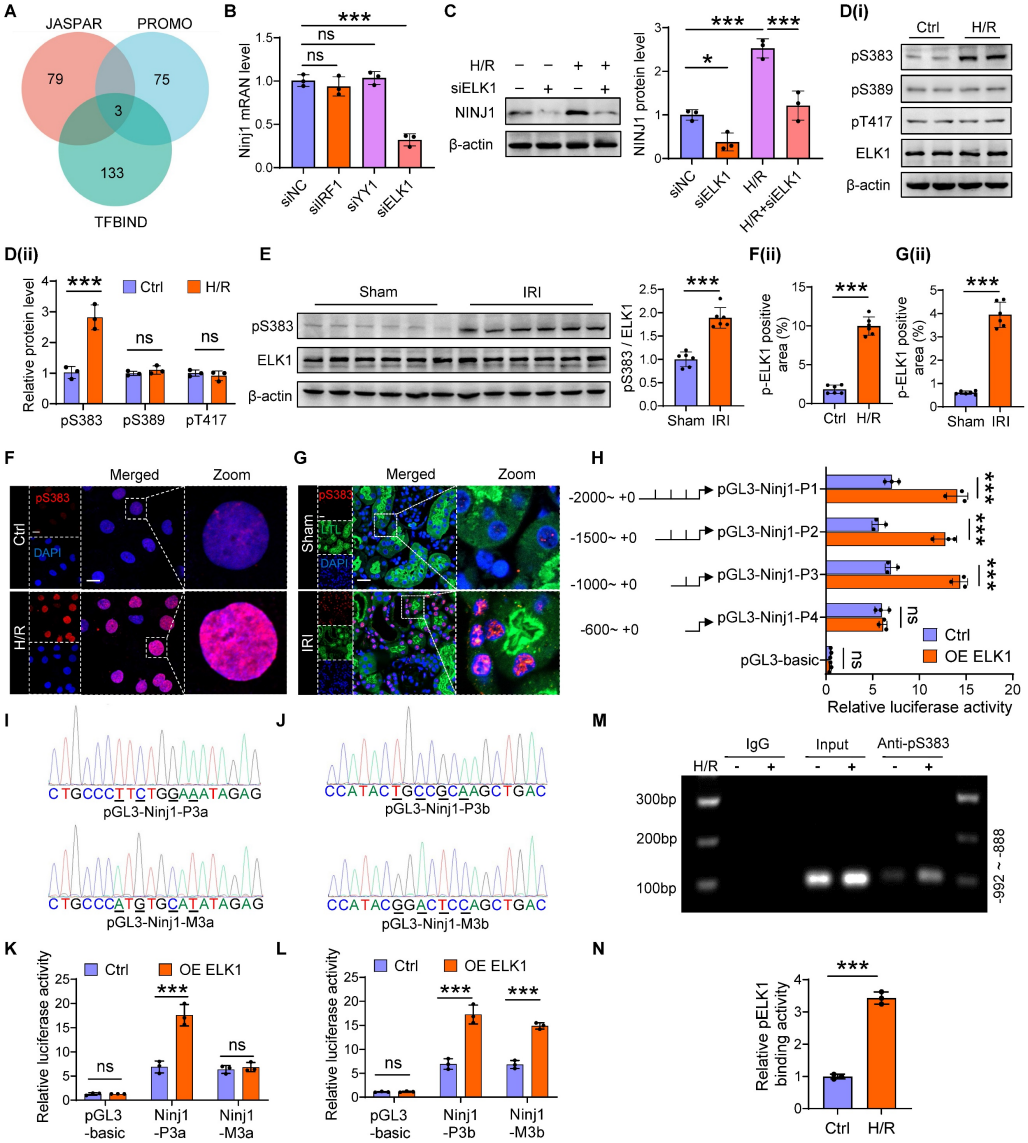
** ELK1 transcriptionally upregulates NINJ1 expression by directly binding to NINJ1 promoter. (A)** JASPAR, PROMO and TFBIND were employed to predict the upstream transcription factors of Ninj1, and three potential transcription factors were obtained, including interferon regulatory factor 1 (IRF1), Yin-Yang 1 (YY1) and ETS transcription factor (ELK1). **(B)** The mRNA level of Ninj1 in HK-2 cells transfected with indicated siRNA (n = 3). **(C)** Western blot analysis of NINJ1 expression in HK-2 cells transfected with siELK1 under normoxia or H/R conditions (n = 3).** (D)** Western blot analysis of p-ELK1 (S383), p-ELK1 (S389), p-ELK1 (T417), and ELK1 expression in HK-2 cells under normoxia or H/R conditions (n = 3).** (E)** Relative expression of p-ELK1 (S383) and ELK1 in renal tissues of sham and IRI-induced AKI mice (n = 6). **(F, G)** Representative immunofluorescence staining and quantification of p-ELK1 (S383) in HK-2 cells (F) and renal tissues (G). Scale bar = 50 μm. **(H)** The pRL-TK vector and pGL3-basic or recombinant reporter plasmids containing various fragments of Ninj1 promoter region were co-transfected in HK-2 cells with or without ELK1 overexpression, and then the cells were collected for dual-luciferase reporter assay. **(I, J)** The pGL3-Ninj1-P3a, pGL3-Ninj1-P3b, pGL3-Ninj1-M3a, and pGL3-Ninj1-M3b fragments (mutant bases are signified by underlines in the sequencing results).** (K, L)** Luciferase assay of HK-2 cells co-transfected with pRL-TK and indicative plasmid in **I** and **J** with or without ELK1 overexpression.** (M, N)** ChIP assay of p-ELK1 (S383) and Ninj1 in HK-2 cells under normoxia or H/R conditions. IgG as a negative control. The PCR and qPCR analyses are respectively shown in **M** and **N** (n = 3). Data are shown as mean ± SD. *P < 0.05; ***P < 0.001. ns: no significance.

Then, we next sought to investigate the molecular mechanism underlying the increased expression of NINJ1. Intriguingly, H/R had no significant effect on the mRNA stability and protein degradation of NINJ1 (Supplementary Figure 6A, B). In parallel, bioinformatic analysis predicted the potential transcription factors of NINJ1 based on three distinct databases, identifying IRF1, YY1 and ELK1 as the promising candidates (Figure 6A). Notably, knockdown of ELK1, instead of IRF1 and YY1, dramatically downregulated the transcription level of NINJ1 (Figure 6B, C and Supplementary Figure 6C). ELK1 is a member of the ETS family of transcription factors, which play an important role in regulating cell proliferation and death 30. In particular, phosphorylation of ELK1 is regarded as a key step for its activation and subsequent nuclear translocation 31. Accordingly, we detected that the total ELK1 expression remained almost unchanged, but ELK1 phosphorylation at Ser^383^ site instead of Ser^389^ and Thr^417^ site, the classical phosphorylation sites of ELK1 31, was elevated after H/R *in vitro* and IRI* in vivo* (Figure 6D, E), along with increased nuclear translocation (Figure 6F, G), whereas ELK1 knockdown significantly diminished ELK1 phosphorylation at Ser^383^ site (Supplementary Figure 6D). These lines of evidence suggest that ELK1 activation contributes to the upregulation of NINJ1. Furthermore, we constructed a dual luciferase reporter system to identify the targeting efficacy of ELK1 on NINJ1. Interestingly, overexpression of ELK1 markedly increased luciferase activity of pGL3-NINJ1-P1~P3, while no effect was observed on pGL3-NINJ1-P4 (Figure 6H and Supplementary Table 6), demonstrating that the binding site is located within the sequence -1000 to -600 relative to the transcription start site. Bioinformatic analysis predicted that there are two binding sites, pGL3-Ninj1-P3a and pGL3-Ninj1-P3b, situated between the sequence -1000 to -600 (Figure 6I, J). Based on this prediction, we constructed these two mutant reporter plasmids, and found that mutation of pGL3-Ninj1-P3a abolished the effect of ELK1 overexpression on increased luciferase activity (Figure 6K, L). Besides, using ChIP, we also observed that Ser^383^-phosphorylated ELK1 could directly bind to the promoter of NINJ1, and their binding was further enhanced after H/R (Figure 6M, N and Supplementary Table 7). Taken together, Ser^383^ phosphorylation of ELK1 could regulate NINJ1 transcription expression by directly binding to the NINJ1 promoter after AKI.

### ELK1 mutation at serine 383 (Ser^383^) phosphorylation mitigates NINJ1-induced inflammatory response

**Figure 7:**
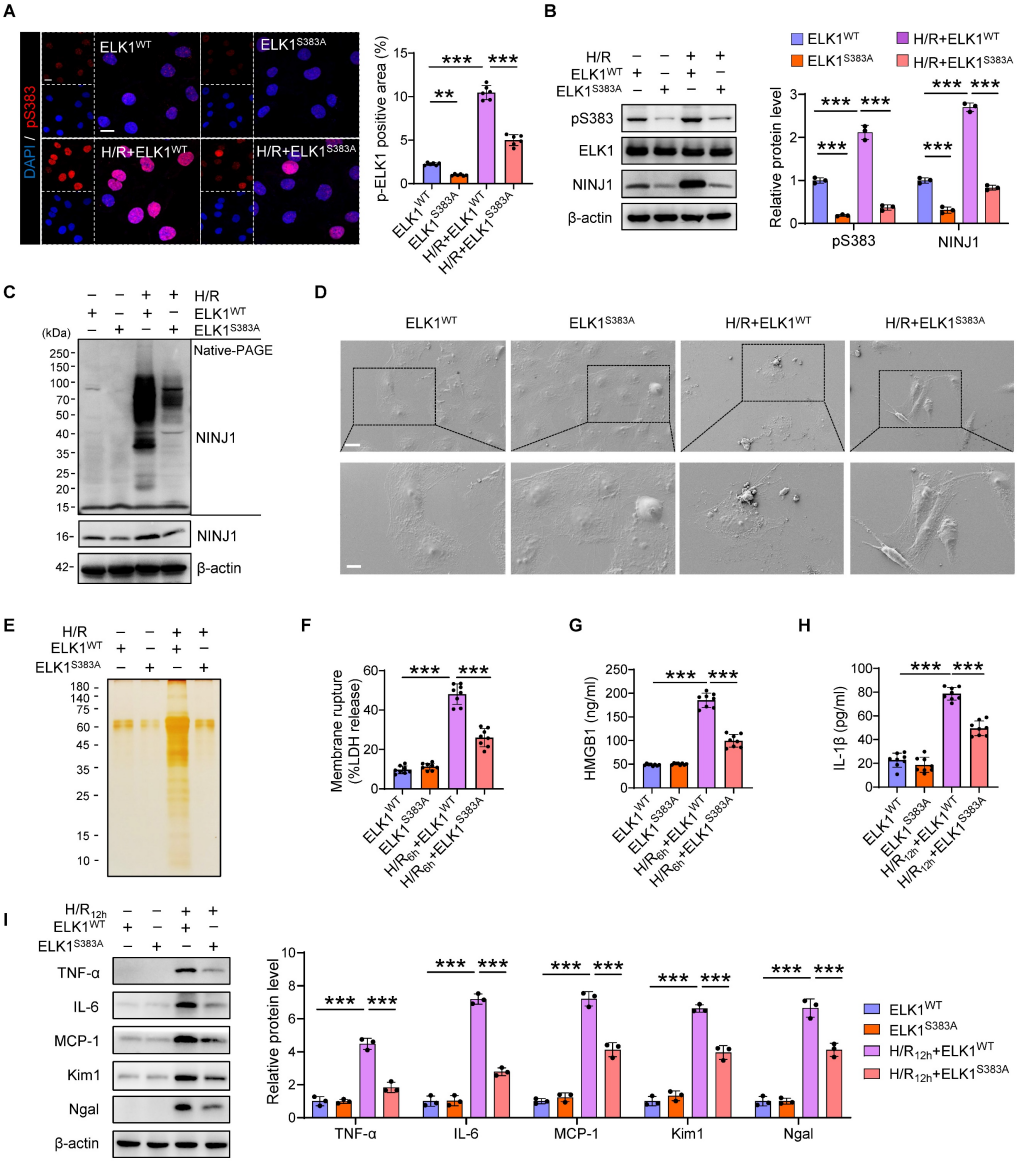
** ELK1 mutation at serine 383 (Ser^383^) phosphorylation mitigates NINJ1-induced inflammatory response.** HK-2 cells were transfected with ELK1^WT^ plasmid or ELK1^S383A^ plasmid under normoxia or H/R conditions.** (A)** Representative immunofluorescence staining and quantification of p-ELK1 (S383) in HK-2 cells following the described treatment (n = 6). Scale bar = 50 μm. **(B)** Western blot analysis of p-ELK1 (S383), ELK1 and NINJ1 expression in HK-2 cells following the described treatment (n = 3). **(C)** Native-PAGE analysis of endogenous NINJ1 in HK-2 cells following the described treatment. **(D)** SEM observation of HK-2 cells following the described treatment. Scale bar = 20 μm (top); 10 μm (down).** (E)** Silver staining of released proteins in culture supernatant of HK-2 cells following the described treatment.** (F-H)** Release of LDH (F) and HMGB1 (G) in culture supernatant of HK-2 cells after 6 hours of reoxygenation and IL-1β (H) at 12 hours after reoxygenation (n = 8). **(I)** Western blot analysis of TNF-α, IL-6, MCP-1, *Kim1*, and *Ngal* in HK-2 cells following by 12 hours of reoxygenation (n = 3). Data are shown as mean ± SD. **P < 0.01, ***P < 0.001.

To further identify the role of Ser^383^ phosphorylation site of ELK1 in NINJ1-indued DAMPs release and inflammatory response, we mutated the Ser^383^ residue and transfected the mutant into HK-2 cells. Indeed, the significantly increased phosphorylation of ELK1 at Ser^383^ and nuclear translocation were dramatically abolished by mutation of Ser^383^, as well as NINJ1 expression (Figure 7A, B), indicating that Ser^383^-phosphorylation of ELK1 play a crucial role in NINJ1 transcription regulation. Moreover, NINJ1 oligomerization was inhibited in HK-2 cell with Ser^383^ mutation of ELK1 (Figure 7C), thereby retaining the ballooning morphology rather than bubble disintegration (Figure 7D). Besides, a pronounced decrease in LDH, HMGB1, IL-1β, and other proteins was found in the supernatant of cells with the Ser^383^ mutation of ELK1 (Figure 7E-H), ultimately alleviating IRI-induced RTEC injury and inflammatory responses (Figure 7I and Supplementary Figure 7A-E). Hence, these data demonstrate that targeting Ser^383^-phosphorylation of ELK1 may restrict NINJ1-induced inflammatory response and renal injury.

### Targeting ELK1 Ser^383^ phosphorylation counteracts NINJ1-induced inflammation after AKI

**Figure 8:**
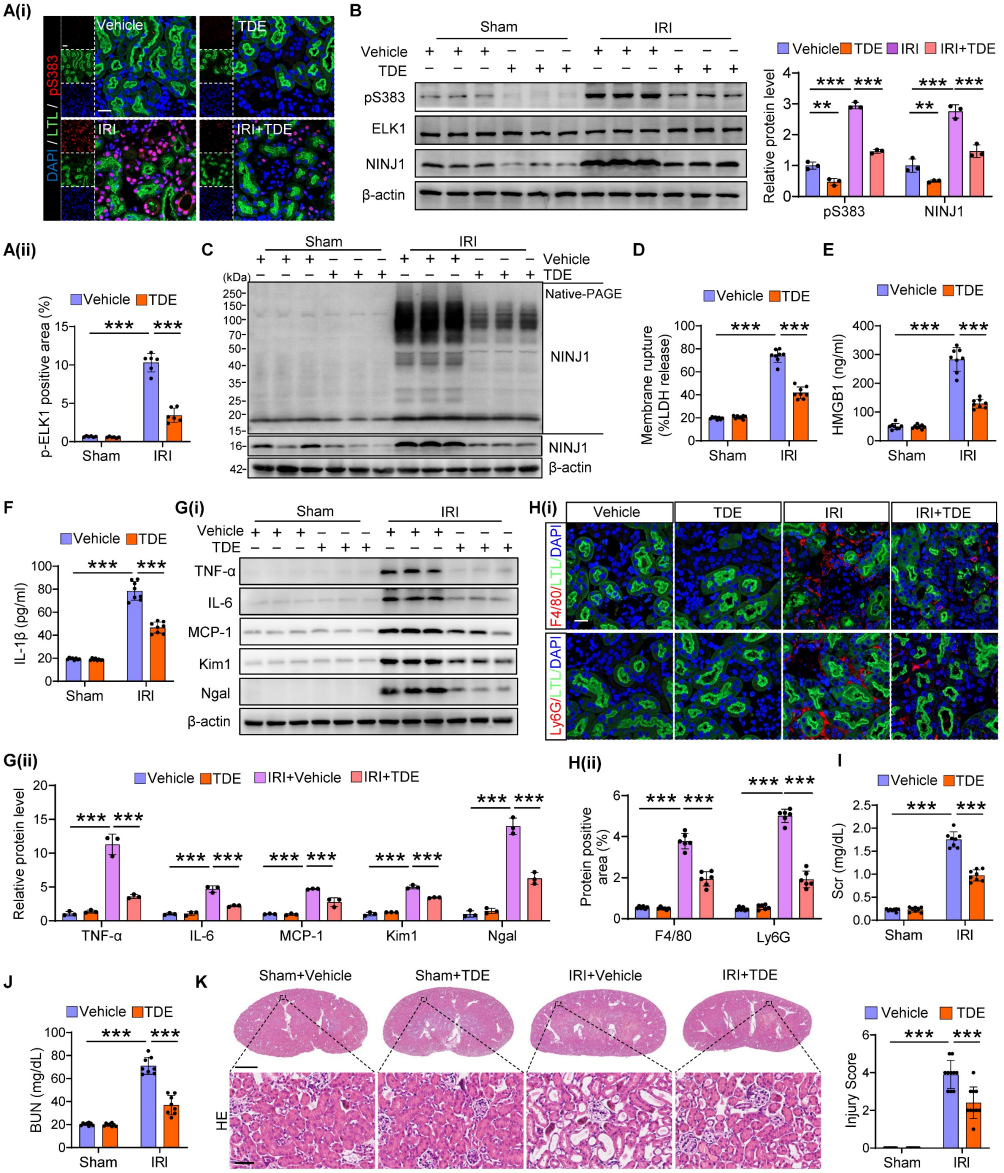
** Targeting ELK1 Ser^383^ phosphorylation counteracts NINJ1-induced inflammation after AKI.** Mice were intraperitoneally injected with vehicle or TDE (2 mg/kg) before subjection to sham or IRI. **(A)** Representative immunofluorescence staining and quantification of p-ELK1 (S383) in renal tissues from mice following the described treatment (n = 6). Scale bar = 50 μm. **(B)** Western blot analysis of p-ELK1 (S383), ELK1 and NINJ1 expression in renal tissues from mice following the described treatment (n = 3). **(C)** Native-PAGE analysis of endogenous NINJ1 in renal tissues following the described treatment. **(D-F)** Release of LDH (D), HMGB1 (E) and IL-1β (F) in serum from mice following the described treatment (n = 8).** (G)** Western blot analysis of TNF-α, IL-6, MCP-1, *Kim1*, and *Ngal* expression in renal tissues (n = 3).** (H)** Confocal microscopy showing the expression of F4/80 or Ly6G in renal tissues from mice following the described treatment (n = 6). Scale bar = 50 μm.** (I, J)** Levels of Scr (I) and BUN (J) of the renal tissues from mice following the described treatment (n = 8). **(K)** Hematoxylin and eosin (HE) staining and injury score in renal tissues from mice following the described treatment (n = 8). Scale bars = 1.25 mm (top); 50 μm (down). Data are shown as mean ± SD. **P < 0.01; ***P < 0.001.

Finally, we utilized a cell-penetrating peptide (TAT-DEF-ELK1 peptide; TDE), which has been shown to inhibit ELK1 phosphorylation at Ser^383^ 32, to protect against inflammation induced by NINJ1 transcriptional upregulation. First, we identified that a high dose of TDE, intraperitoneally injected into mice, did not cause significant pathological injury to the heart, liver, spleen, lung, kidney or intestine, nor did it affect blood routine, and liver and kidney function (Supplementary Figure 8A-M). In addition, no toxicity was observed in HK-2 cells following treatment with TDE up to the concentration of 50 μM (Supplementary Figure 9A). These *in vivo* and *in vitro* results suggest the superior biosafety of TDE. Consistently, TDE treatment significantly inhibited ELK1 phosphorylation at Ser^383^ induced by H/R *in vitro* and IRI *in vivo* (Figure 8A, B and Supplementary Figure 9B-D). NINJ1 expression and oligomerization, as well as the release of LDH and DAMPs were accordingly alleviated by TDE treatment both *in vivo* (Figure 8B-F) and *in vitro* (Supplementary Figure 9E-I), accompanied by a decrease in pro-inflammation factors (Figure 8G and Supplementary Figure 10A-I), immune cells infiltration (Figure 8H and Supplementary Figure 11A), as well as pro-inflammation phenotype macrophage activation (Supplementary Figure 11B-D). Consequently, TDE treatment safeguard IRI-induced kidney injury, as evidenced by improved morphology of injury and lower levels of *Kim1* and *Ngal*, along with the preservation of kidney functions (Figure 8G, I-K and Supplementary Figure 10J, K). These findings suggest that TDE may serve as a promising therapeutic drug for alleviating NINJ1-mediated sterile inflammation through targeting Ser^383^-phosphorylation ELK1.

## Discussion

NINJ1 was identified as an executor of plasma membrane rupture during necroptosis, pyroptosis and secondary necrosis, and these pathologies co-exist within the AKI patients as well. However, few studies have focus on the relationship between NINJ1 and AKI. Here, we uncover that NINJ1 expression significantly increased in RTECs in response to distinct AKI stimuli. Functionally, NINJ1 oligomerizes and promotes plasma membrane rupture, thereby releasing DAMPs and amplifying the inflammatory response. Importantly, we identify that ELK1 is phosphorylated at Ser^383^, nuclear translocation, and regulates NINJ1 transcription. Therapeutically, genetic knockout of NINJ1 or pharmacological inhibition of ELK1 phosphorylation effectively protect against AKI and improve AKI prognosis.

Tubular necrosis and nephron loss are common features in a variety of AKI, while the massive changes in morphology imply the death of tubular cells 33. Currently, substantial evidence has reported that several distinct cell death models exist after AKI 34. Non-lytic cell death (apoptosis) and lytic cell death (ferroptosis, pyroptosis, and necroptosis) can be classified among the many types of cell death 35. However, the apoptotic cells will also undergo plasma membrane rupture due to defects in the efferocytosis machinery or massive apoptosis 36. Ultimately, these cells may lose plasma membrane integrity and release DAMPs to cause further inflammation and oxidative stress, resulting in increased the risk of death in patients with AKI 37. Although accumulating studies, including our own, have identified numerous cell death regulators 25, 38, this does not fully address the therapeutic needs of AKI due to the coexistence of cell death models. In this study, we demonstrate that NINJ1, the executor of plasma membrane rupture, was significantly induced in RTECs after AKI. On the contrary, knockdown of NINJ1 in RTECs potently prevents plasma membrane rupture and DAMPs release, further protecting renal inflammation and improving AKI prognosis. Because NINJ1 plays an important role in distinct models of cell death commonly present in AKI patients, it is reasonable to expect that NINJ1 may be a valuable therapeutic target for AKI.

As known, NINJ1 is a 16 kDa plasma membrane protein known to be involved in the inflammatory response, because of its ability to regulate myeloid cell recruitment 39, 40. In living cells, NINJ1 is monomeric in the cellular membrane, whereas upon cell death induction, NINJ1 forms different-sized oligomers to promote membrane rupture through capping membrane edges. Here, *in vivo* and *in vitro* results reveal that NINJ1 undergoes oligomerization after AKI stimuli, thereby inducing plasma membrane rupture. Notably, the increased plasma membrane permeabilization induced by other lytic cell death activators, such as mixed lineage kinase domain like protein (MLKL) and gasdermin D (GSDMD), differs from NINJ1-induced plasma membrane rupture 41. As reported, the pore size induced by MLKL and GSDMD is relatively small, which can only release small molecules, while the release of large DAMPs requires the complete disintegration plasma membrane. Meanwhile, we demonstrate that LDH and HMGB1 were increased in the culture supernatant. Knockdown of NINJ1 significantly alleviated the release of LDH and HMGB1, but not IL-1β, after 6 hours of H/R, which may be attribute to IL-1β be released through MLKL or GSDMD pores. However, we detect a reduction in IL-1β at H/R 12 hours, possibly due to diminished DAMP-mediated inflammatory damage. Taken together, these lines of evidence argue that NINJ1 may play a directly role in the regulation of plasma membrane rupture and further cellular inflammatory damage in RTECs after AKI. In addition, glycine has been reported to prevent plasma membrane rupture through inhibiting NINJ1 oligomerization 29, and this effect was also found in an *in vitro* AKI model, as we revealed here. However, the effect glycine in the AKI mice, reported in a rather limited number of publications, it remains controversial. Almeida et al. reveal that glycine treatment could remain protective at low levels in the proximal tubular injection 42, while another study show that glycine could aggravate AKI through activating N-Methyl-D-Aspartate receptor 43. Hence, further research is needed on the role of glycine in targeting NINJ1 *in vivo*. Since the transcriptional level of NINJ1 is elevated in RTECs after AKI, targeting its transcriptional regulation deserves further study. Based on our findings, we predicted putative transcription factors of NINJ1 using bioinformatics analysis and for the first time identify that ELK1 transcriptional regulation NINJ1 is the main cause for its increased expression after AKI. ELK1 is a member of the ETS transcription factor family and plays a pivotal role in regulating the transcription of genes involved in cell proliferation, differentiation and death 30. Notably, ELK1 is a component of the MAPK pathway and can be activated through reactive oxygen species (ROS)/MAPK signaling, which was highly upregulated in RTECs upon AKI 37, 44. In the present study, we demonstrate ELK1 phosphorylation at Ser^383^ site, subsequent nuclear translocation, and binding with Ninj1 promoter region (-908 to -899, CTTCTGGAAA). Ser^383^ is a potential phosphorylation site that is particularly important for the transactivating effect of ELK1, as a single mutation of this residue induces a complete loss of this effect 31, 45. Besides, since ELK1 has been shown to regulate inflammation in acute lung injury and diabetic nephropathy 46, 47, it is plausible that ELK1 may also be involved in RTEC death and interstitial inflammation. Interestingly, mutation of Ser^383^ or use of TDE can block ELK1 transcriptional activity and prevent plasma membrane rupture. TDE is a cell-penetrating peptide that mimics the DEF domain, which is the major motif for Ser^383^ phosphorylation 48. In addition, the effect of inhibiting Ser^383^ phosphorylation is specific because it does not interfere with MAPK signaling activation and nuclear translocation and, as we revealed here, is biologically safe with few side effects. Hence, it is conceivable that use of this drug may be a promising avenue to prevent plasma membrane rupture in RTECs and renal inflammation in AKI patients.

## Conclusions

Our study shows that increased NINJ1 expression in RTECs underlies plasma membrane rupture and renal inflammatory response after AKI, and demonstrates Ser^383^-phosphorylated ELK1 as a novel transcriptional regulator in NIJI1 expression. These findings not only provide deep insight into the pathogenesis of AKI, but also indicate that targeting the ELK1- NINJ1 signaling axis may be a novel strategy to prevent this pathological process and improve AKI prognosis.

## Supplementary Material

Supplementary figures and tables.

## Abbreviations

NINJ1: nerve injury-induced protein 1
DAMPs: Damage-associated molecular patterns
AKI: acute kidney injury
RTECs: renal tubular epithelial cells
ELK1: ETS transcription factor
CKD: chronic kidney disease
ATN: acute tubular necrosis
Scr: serum creatinine
BUN: blood urea nitrogen
IRI: ischemia-reperfusion injury
Cis: cisplatin
FA: folic acid
LDH: lactate dehydrogenase
H/R: hypoxia/reoxygenation
siNinj1: siRNA against NINJ1
*Kim1*: kidney injury molecule 1
*Ngal*: neutrophil gelatinase-associated lipocalin
TDE: TAT-DEF-ELK1 peptide
AAV9-shNinj1: adeno-associated virus serotype 9-short hairpin Ninj1 plasmid
HMGB1: high mobility group box 1
IL-1β: interleukin 1β
TNF-α: tumor necrosis factor alpha
IL-6: interleukin 6
MCP-1: monocyte chemotactic protein 1
HK-2 cell: human kidney 2 cell
FBS: fetal bovine serum
IRF1: interferon regulatory factor 1
YY1: Yin-Yang 1
PBS: phosphate buffer saline
SEM: scanning electron microscope
SD: standard deviation
THP-1 cell: human acute monocytic leukemia cell
MLKL: mixed lineage kinase domain like protein
GSDMD: gasdermin D
α-SMA: α smooth muscle actin
FN: fibronectin
