# Supplementary material for: Targeting NINJ1-Mediated Plasma Membrane Rupture in Tubular Epithelial Cell Prevents Inflammatory Response in Acute Kidney Injury
Source: Int J Biol Sci. 2025 Aug 11;21(12):5223–39. doi: 10.7150/ijbs.115364 (PMC12435343; doi:10.7150/ijbs.115364)
Supplement: Supplementary file 1 — Supplementary figures and tables. [file ijbsv21p5223s1.pdf]

Supplementary Material

Supplementary Methods

Supplementary Figure 1. NINJ1 expression is highly induced in AKI.

Supplementary Figure 2. Knockdown of Ninj1 mitigates inflammatory response in HK-2 cells.

Supplementary Figure 3. Inhibition of NINJ1 oligomerization alleviate inflammation.

Supplementary Figure 4. Silencing of NINJ1 protects against AKI and improves AKI prognosis.

Supplementary Figure 5. Ninj1-mediate DAMP release in tubular epithelial cells induce the recruitment and activation of macrophages.

Supplementary Figure 6. ELK1 transcriptionally upregulates NINJ1 expression by directly binding to NINJ1 promoter.

Supplementary Figure 7. ELK1 mutation at serine 383 (Ser^{383}) phosphorylation mitigates NINJ1-induced inflammatory response.

Supplementary Figure 8. *In vivo* Toxicity Assessment of TDE.

Supplementary Figure 9. Targeting ELK1 Ser^{383} phosphorylation by TDE treatment counteracts NINJ1-induced inflammation after AKI.

Supplementary Figure 10. Targeting ELK1 Ser^{383} phosphorylation by TDE treatment counteracts NINJ1-induced inflammation after H/R *in vitro* and IRI *in vivo*.

Supplementary Figure 11. TDE treatment counteracts Ninj1-mediate DAMP release in tubular epithelial cells induce the recruitment and activation of macrophages.

Supplementary Table 1: Clinical data of ATN and non-ATN patients examined.

Supplementary Table 2: The sequence sets for siRNA.

Supplementary Table 3: Primary Antibody list.

Supplementary Table 4: The primer sets for human.

Supplementary Table 5: The primer sets for mouse.

Supplementary Table 6: Putative binding sequences of ELK1 in Ninj1 promoter region.

Supplementary Table 7: The primer sets for ChIP.

Supplementary Methods

Cell viability assay

Cell viability was assessed using the Cell Counting Kit-8 (CCK-8, HY-K0301, MedChemExpress, New Jersey, USA). After TAT-DEF-ELK1 peptide (TDE, HY-P2262, MedChemExpress) treatment of human kidney 2 (HK-2) cells for 24 hours, cells were incubated with 100 μl of culture medium containing 10 μl of CCK-8 solution at 37 °C for 2 h and then the absorbance was measured at 450 nm by a SpectraMAX M3 microplate reader.

Toxicity assessment

C57BL/6J mice were intraperitoneally injected with different doses of TDE (control, 2mg/kg, 5mg/kg, and 10mg/kg). After 30 days, mice were sacrificed. Blood samples were collected for hematology, liver and kidney function tests. The degree of injury to the heart, liver, spleen, lungs, kidneys, and intestines were analyzed by hematoxylin and eosin (HE) staining [1].

Scr and BUN measurements

The mouse blood samples were collected and centrifuged at 3000 rpm for 15 min, and then upper serum were collected. The levels of serum creatinine (Scr) and blood urea nitrogen (BUN) were detected by urea and creatinine assay kit (Nanjing Jiancheng Bioengineering Institute, Nanjing, China) according to the manufacturer’s instructions.

Histology

The kidney tissues were fixed in 4% paraformaldehyde and cut into 2.5 μm-thickness slices. The sections were stained with HE, Masson and were photographed under a

microscope (Olympus Optical DP70, Tokyo, Japan).

Immunofluorescence

The sections were incubated with primary antibodies overnight at 4°C, followed with Cy3 or FITC-coupled secondary antibodies or LTL (FL-1321, Vector Laboratories, San Francisco, California, USA) at 37 °C for 1 hour. Then incubated with DAPI (C1006, Beyotime Biotechnology, Shanghai, China) for 5 minutes, tissues were photographed under the Zeiss LSM900 NLO confocal microscope. The quantification was performed according to positive area or fluorescence intensity of proteins using ImageJ software.

Immunohistochemistry

De-paraffinized sections following antigen retrieval with citrate buffer above 95 °C for 30 minutes were incubated with 0.3% H_2O_2 at room temperature for 15 minutes. Followed by blocking with goat serum, sections were stained with primary antibodies at 4 °C overnight, and was performed using the IHC Assay kit (ZSGB-BIO, Beijing, China). Nuclei were stained by hematoxylin. As described previously, renal tubular immunostaining for NINJ1 in biopsy specimens was independently quantified by two investigators in a blinded manner using a semi-quantitative scoring system (0-4): (score 0: absence of specific staining; score 1: <25% area has specific staining for NINJ1; score 2: 25%–50%; score 3: 50%–75%; score 4: >75%) [2].

Renal tubular injury score evaluation

The tubular injury score was evaluated by two independent pathologists from 10 randomly selected fields from each renal tissue stained with HE. Two independent pathologists assessed the severity of renal tubule injury based on the percentage of

damaged tubules. The score criterion was as follows, 0: normal; 1: mild injury, involvement of 0% - 10%; 2: moderate injury, involvement of 11% - 25%; 3: severe injury, involvement of 26% - 49%; 4: high severe injury, involvement of 50% - 75%; 5: extensive injury, involvement of > 75% [3]. All assessments were done blindly.

Western blot.

The proteins from renal cortexes or cells were extracted with RIPA lysis buffer (P0013, Beyotime Biotechnology) containing protease and phosphatase inhibitor cocktail (Roche Diagnostics GmbH, Mannheim, Germany), and the concentration was determined by the BCA kit (P00009, Beyotime Biotechnology). Protein samples were separated by SDS-PAGE gel and transferred to PVDF membranes (Merck Millipore, Billerica, MA, USA). After blocked by QuickBlock blocking buffer (P0252, Beyotime Biotechnology) at 37 °C for 30 min, the membranes were separately incubated with primary antibodies at 4 °C overnight. Then membranes were incubated with the corresponding HRP-conjugated secondary antibodies (Beyotime Biotechnology) for 1 hour at 37 ℃. Subsequently, the signals were detected by ECL chemiluminescence reagent (ProteinSimple, Santa Clara Valley, CA, USA). The primary antibodies were listed in Supplementary Table 3. Grayscale results were analyzed by ImageJ software.

Construction of reporter plasmids and point mutation.

Putative ELK1 binding sites in the Ninj1 promoter region are listed in Supplementary Table 6. Various lengths of the Ninj1 promoter region were amplified by PCR using the genomic DNA of HK-2 cells as a template. The fragments including Ninj1-2000 (−2000 to +0), Ninj1-1500 (−1500 to +0), Ninj1-1000 (−1000 to +0) and Ninj1-600 (−600 to

+0) were separately cloned into a pGL3-basic vector (Promega, Madison, Wisconsin, USA) after digestion with HindIII, and the recombinant reporter plasmids were separately named as pGL3-Ninj1P1, pGL3-Ninj1P2, pGL3-Ninj1P3 and pGL3-Ninj1P4. The mutant plasmids pGL3-Ninj1-M3a and pGL3-Ninj1-M3b containing point mutations in the ELK1 binding element (CTGCCCATGTGCATATAGAG, CCATACGGACTCCAGCTGAC, respectively, the mutated bases are underlined) were generated with MutanBEST kit (Takara, Tokyo, Japan) using pGL3-Ninj1-P3 (−1000 to +0) as a template.

Luciferase reporter constructs and dual-luciferase reporter assay

The recombinant reporter plasmids were co-transfected with pcDNA3.1 vector (Promega, Madison, USA) or ELK1 overexpression plasmids and Renilla plasmids into HK-2 cells using Lipofectamine 3000. Luciferase activity was detected using The Dual-Luciferase Reporter Assay System (E1910, Promega). Firefly luciferase activity was normalized against Renilla activity.

Chromatin immunoprecipitation (ChIP)

ChIP assays were performed by using a Simple Enzymatic ChIP Kit (26157, Invitrogen) according to the manufacturer's instructions. After treatment, HK-2 cells were incubated with 1% formaldehyde for crosslinking. Next, cells were lysed in sodium dodecyl sulfate lysis buffer containing a protease/phosphatase inhibitor. The resulting chromatin was sonicated to shear DNA to an average length between 200 to 1000 bp. The clipped cross-linked chromatin was co-precipitated with anti-p-ELK1 antibody or IgG (as a control) overnight. The harvested chromatin was then washed and incubated

at 65 °C for 30 min with vigorous shaking. DNA Column was used to purify DNA and performed qPCR detection. The primers for ChIP are listed in Supplementary Table 7.

Co-culture Transwell assay

Hypoxia/reoxygenation (H/R) treated HK-2 cells and macrophage co-culture was performed as previously described[4]. Transwell with 0.4 μm and 8 μm pores (Corning, USA) were purchased to demonstrate the process of HK-2 cells communicating with THP-1 macrophages in different states. For soluble factor communication studies, HK-2 cells were seeded in the upper chamber of the Transwell with 0.4 μm pores, and macrophages differentiated from THP-1 cells were seeded in the lower chamber. HK-2 cells were first subjected to hypoxia for 24 hours, and then were reoxygenated and co-cultured with macrophages for the indicated time. For the chemotaxis test, macrophages were cultured in the upper chamber of transwells (8 μm) and HK-2 cells were cultured in the lower chamber. After cocultured for indicated time, the transwells were fixed and stained with 0.1% crystal violet (C0121, Beyotime Biotechnology).

Flow cytometry

macrophages that co-cultured with HK-2 cells were harvested. Cells were stained with antibodies against F4/80-FITC and CD86-eFluor 647 for 30 min. Cells were detected using a BD FACSverse flow cytometer (BD Biosciences). Data analysis was performed using FlowJo software (Tree Star Inc.).

Supplementary References

1. Xin W, Gong S, Chen Y, Yao M, Qin S, Chen J, et al. Self-Assembling P38 Peptide Inhibitor Nanoparticles Ameliorate the Transition from Acute to Chronic Kidney Disease by Suppressing Ferroptosis. Adv Healthc Mater. 2024; 13: e2400441.

2. Fan Y, Xiao W, Lee K, Salem F, Wen J, He L, et al. Inhibition of Reticulon-1A-Mediated

Endoplasmic Reticulum Stress in Early AKI Attenuates Renal Fibrosis Development. J Am Soc Nephrol. 2017; 28: 2007-21.

3. Yang B, Lan S, Dieude M, Sabo-Vatasescu JP, Karakeussian-Rimbaud A, Turgeon J, et al. Caspase-3 Is a Pivotal Regulator of Microvascular Rarefaction and Renal Fibrosis after Ischemia-Reperfusion Injury. J Am Soc Nephrol. 2018; 29: 1900-16.

4. Lv LL, Feng Y, Wen Y, Wu WJ, Ni HF, Li ZL, et al. Exosomal CCL2 from Tubular Epithelial Cells Is Critical for Albumin-Induced Tubulointerstitial Inflammation. J Am Soc Nephrol. 2018; 29: 919-35.

Supplementary Figure

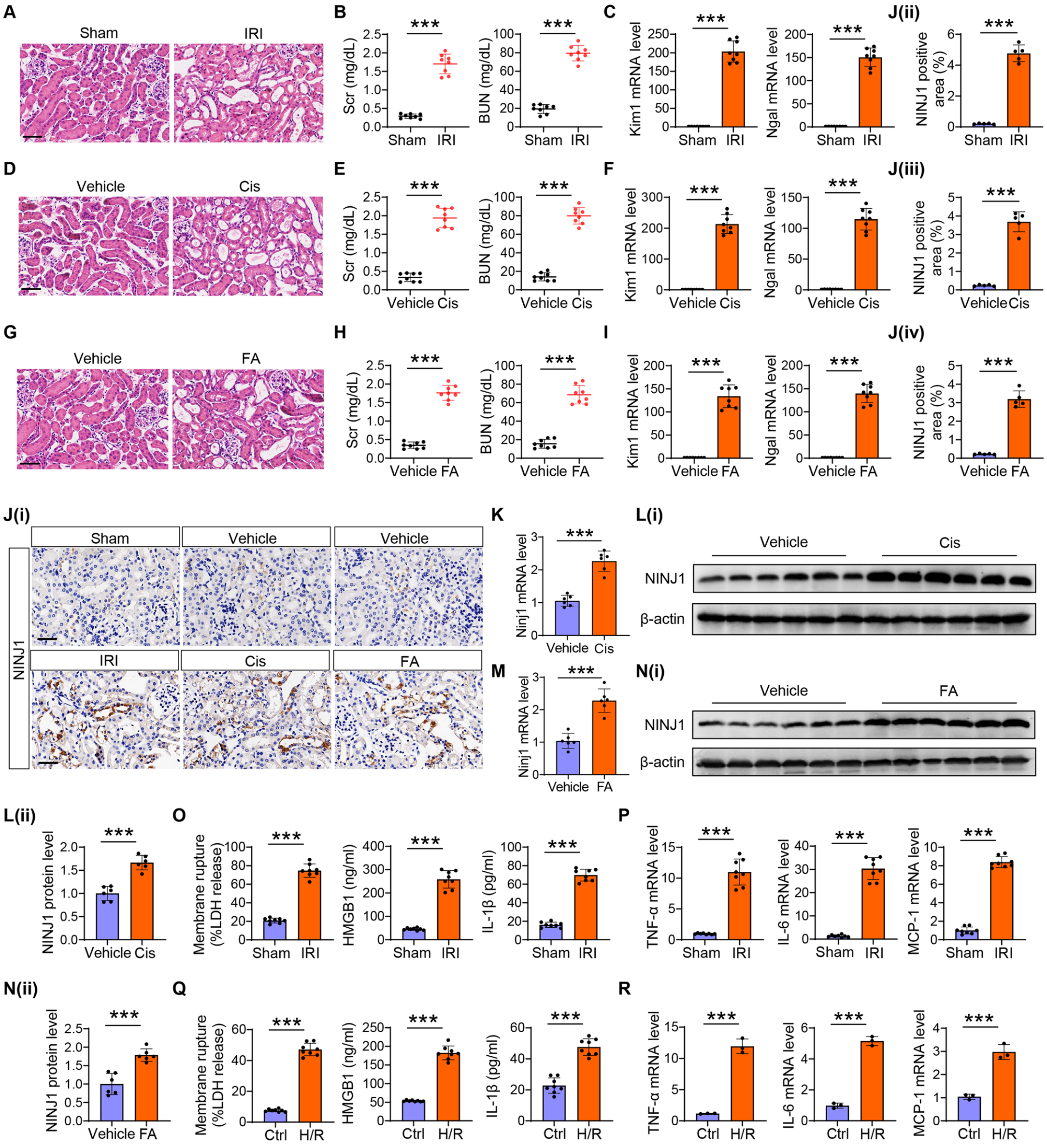

Supplementary Figure 1. NINJ1 expression is highly induced in AKI.

A, D, G Representative hematoxylin and eosin (HE) staining in ischemia-reperfusion injury (IRI)-induced AKI, cisplatin (Cis)-induced AKI and folic acid (FA)-induced AKI. Scale bar = 50 μm. **B, E, H** Serum levels of serum creatinine (Scr) and blood urea nitrogen (BUN) in IRI-induced AKI, Cis-induced AKI and FA-induced AKI (n = 8). **C,**

F, I qPCR analysis of kidney injury molecule 1 (*Kim1*) and neutrophil gelatinase-associated lipocalin (*Ngal*) in IRI-induced AKI, Cis-induced AKI and FA-induced AKI (n = 8). **J** Representative immunohistochemical imaging and quantification of NINJ1 in IRI-induced AKI, Cis-induced AKI and FA-induced AKI (n = 5). Scale bar = 50 μm. **K, L** Expression of NINJ1 in kidneys of sham and Cis-induced AKI mice, determined respectively by qPCR (K) and western blot (L) (n = 6). **M, N** Expression of NINJ1 in kidneys of sham and FA-induced AKI mice, determined respectively by qPCR (M) and western blot (N) (n = 6). **O** Lactate dehydrogenase (LDH), high mobility group box 1 (HMGB1) and interleukin 1β (IL-1β) levels in serum of sham and IRI-induced AKI mice (n = 8). **P** qPCR analysis of tumor necrosis factor alpha (TNF-α), interleukin 6 (IL-6) and monocyte chemotactic protein 1 (MCP-1) in renal tissues of sham and IRI-induced AKI mice (n = 8). **Q** LDH, HMGB1 and IL-1β levels in culture supernatant of human kidney 2 (HK-2) cells under normoxia or hypoxia/reoxygenation (H/R) conditions (n = 8). **R** qPCR analysis of TNF-α, IL-6 and MCP-1 in culture supernatant of HK-2 cells under normoxia or H/R conditions (n = 3). Data are shown as mean ± standard deviation (SD). ***$P < 0.001$.

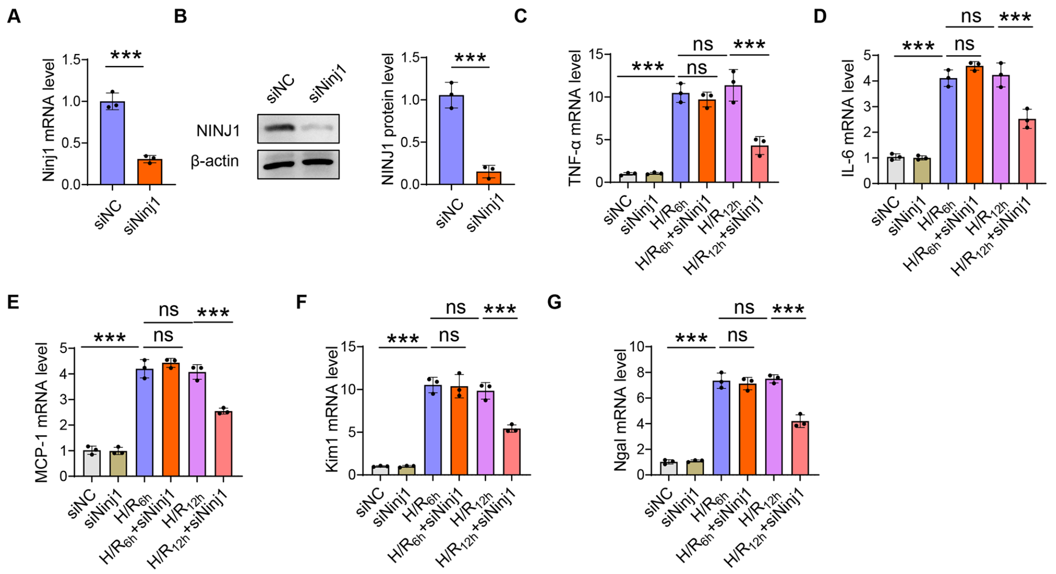

Supplementary Figure 2. Knockdown of Ninj1 mitigates inflammatory response in HK-2 cells.

A, B The qPCR analysis (A) and western blot analysis (B) of NINJ1 in HK-2 cells transfected with siRNA targeting Ninj1 (siNinj1) or non-targeted control (siNC) (n = 3). **C-G** qPCR analysis of TNF-α (C), IL-6 (D), MCP-1 (E), *Kim1* (F), and *Ngal* (G) expression in HK-2 cells after reoxygenation at indicate time (n = 3). Data are shown as mean ± SD. ****P* < 0.001. ns: no significance.

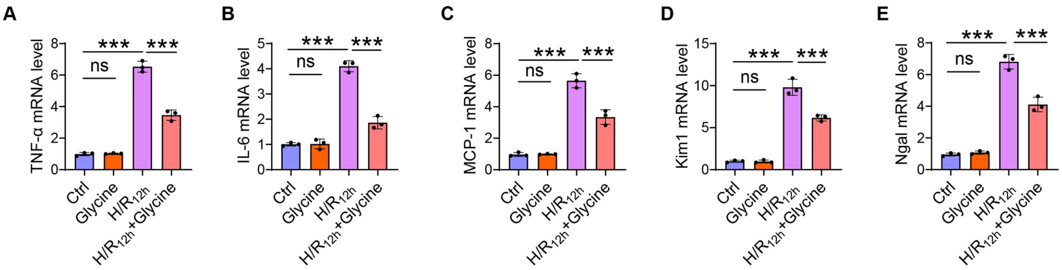

Supplementary Figure 3. Inhibition of NINJ1 oligomerization alleviate inflammation.

A-E qPCR analysis of TNF-α (A), IL-6 (B), MCP-1 (C), *Kim1* (D), and *Ngal* (E) expression in HK-2 cells with or without glycine treatment after reoxygenation at indicate time (n = 3). Data are shown as mean ± SD. ***$P < 0.001$. ns: no significance.

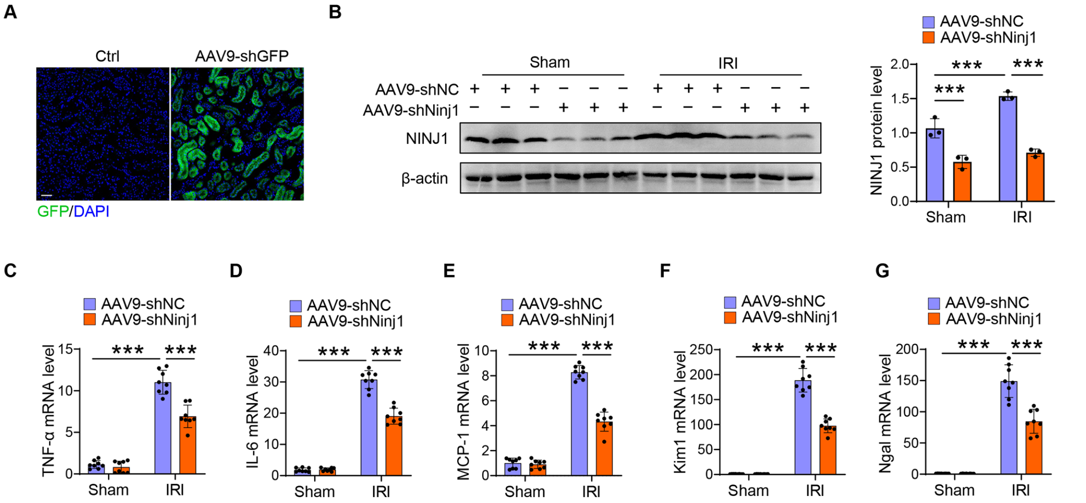

Supplementary Figure 4. Silencing of NINJ1 protects against AKI and improves AKI prognosis.

A, B Representative immunofluorescence staining of GFP in the renal cortex and western blot analysis (n = 3) from AAV9-Ksp-GFP-shNinj1 injection mice. Scale bar = 50 μm. **C-G** qPCR analysis of TNF-α (C), IL-6 (D), MCP-1 (E), *Kim1* (F), and *Ngal* (G) expression in renal tissues from mice with AAV9-shNinj1 or AAV9-shNC administration (n = 8). Data are shown as mean ± SD. ***$P < 0.001$.

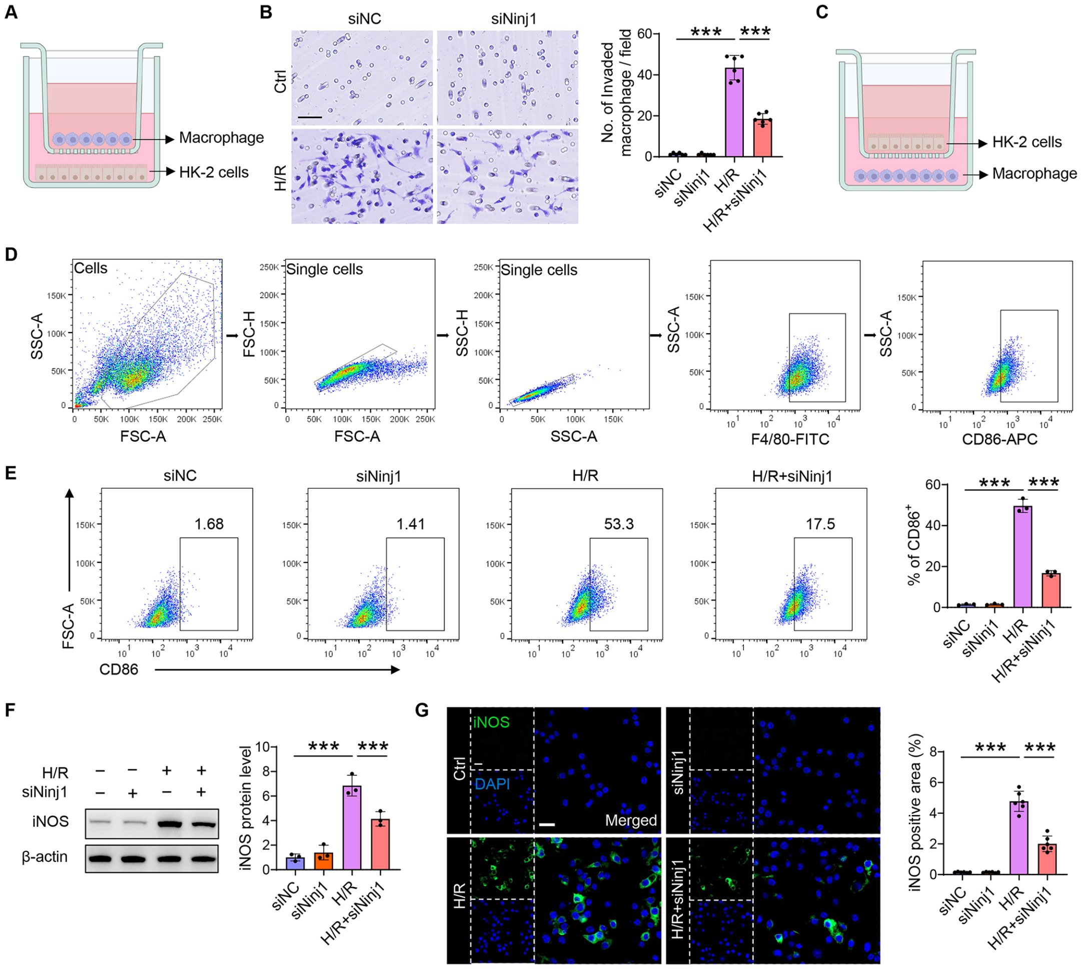

Supplementary Figure 5. Ninj1-mediate DAMP release in tubular epithelial cells induce the recruitment and activation of macrophages.

A An in vitro co-culture system was used in which HK-2 cells were seeded in the bottom compartment, separated by a porous membrane from THP-1 macrophages that were cultured in the top compartment. **B** Representative images and quantification of macrophages stained with crystal violet following the described treatment in A (n = 6). Scale bar = 50 μm. **C** An in vitro co-culture system was used in which HK-2 cells were seeded in the top compartment, separated by a porous membrane from THP-1 macrophages that were cultured in the bottom compartment. **D** Gating strategy used to

identify M1 macrophage (F4/80^{high}CD86^{high}). **E** Representative flow cytometry (FC) analysis of the percentage of M1 macrophage in co-cultured with HK-2 cells following the described treatment in C (n = 3). **F** Western blot analysis of iNOS expression in macrophage following the described treatment in C (n = 3). **G** Representative immunofluorescence staining and quantification of M1 macrophage following the described treatment in C (n = 6). Scale bar = 50 μm. Data are shown as mean ± SD. ***$P < 0.001$.

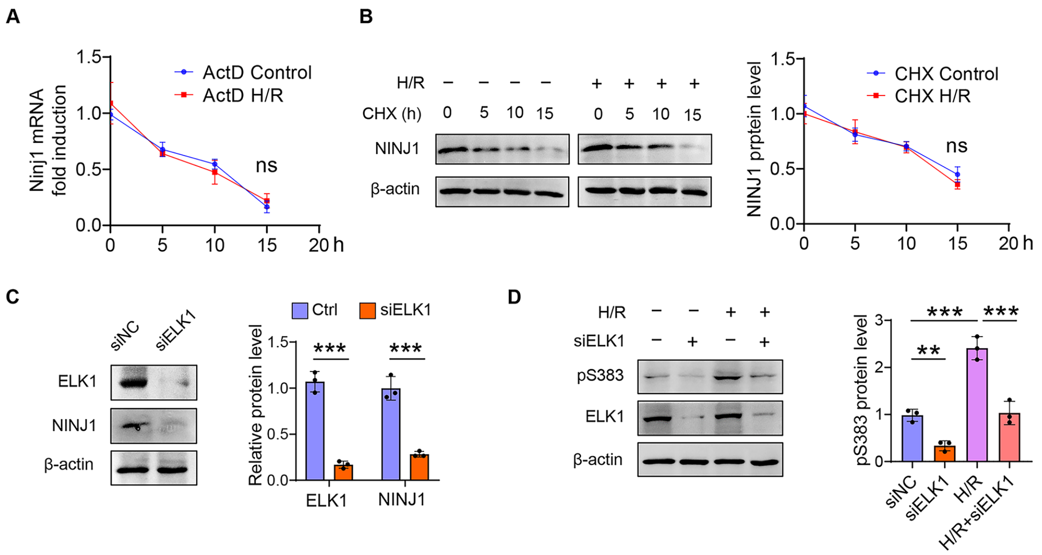

Supplementary Figure 6. ELK1 transcriptionally upregulates NINJ1 expression by directly binding to NINJ1 promoter.

A HK-2 cells were treated with a transcriptional inhibitor actinomycin D (ActD, 0.5 μg/ml) for various time in the absence or presence of H/R. Ninj1 mRNA expression was determined using qPCR (n = 3). **B** Cells were treated with a translational inhibitor cycloheximide (CHX, 10 μM) time-dependently in the absence or presence of H/R. NINJ1 protein expression was detected using western blot (n = 3). **C** Western blot analysis of ELK1 and NINJ1 expression in siELK1-treated cells (n = 3). **D** Western blot analysis of p-ELK1 (S383) and ELK1 expression in siELK1-treated cells under normoxia or H/R conditions (n = 3). Data are shown as mean ± SD. ***P* < 0.01; ****P* < 0.001. ns: no significance.

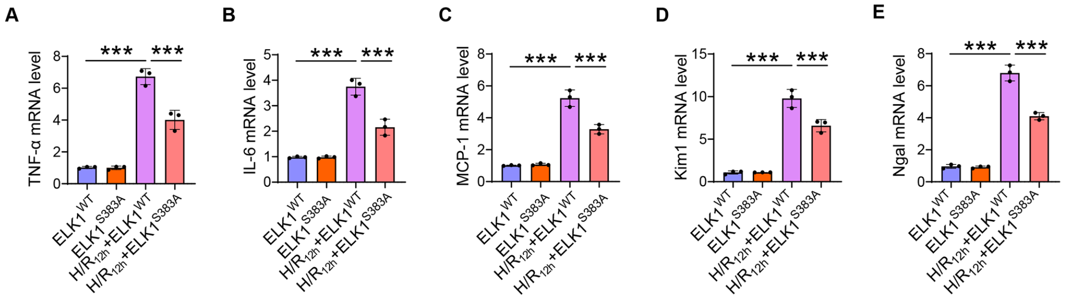

Supplementary Figure 7. ELK1 mutation at serine 383 (Ser^{383}) phosphorylation mitigates NINJ1-induced inflammatory response.

A-E qPCR analysis of TNF-α (A), IL-6 (B), MCP-1 (C), *Kim1* (D), and *Ngal* (E) expression in HK-2 cells transfected with ELK1^{WT} plasmid or ELK1^{S383A} plasmid under normoxia or H/R conditions (n = 3). Data are shown as mean ± SD. ****P* < 0.001.

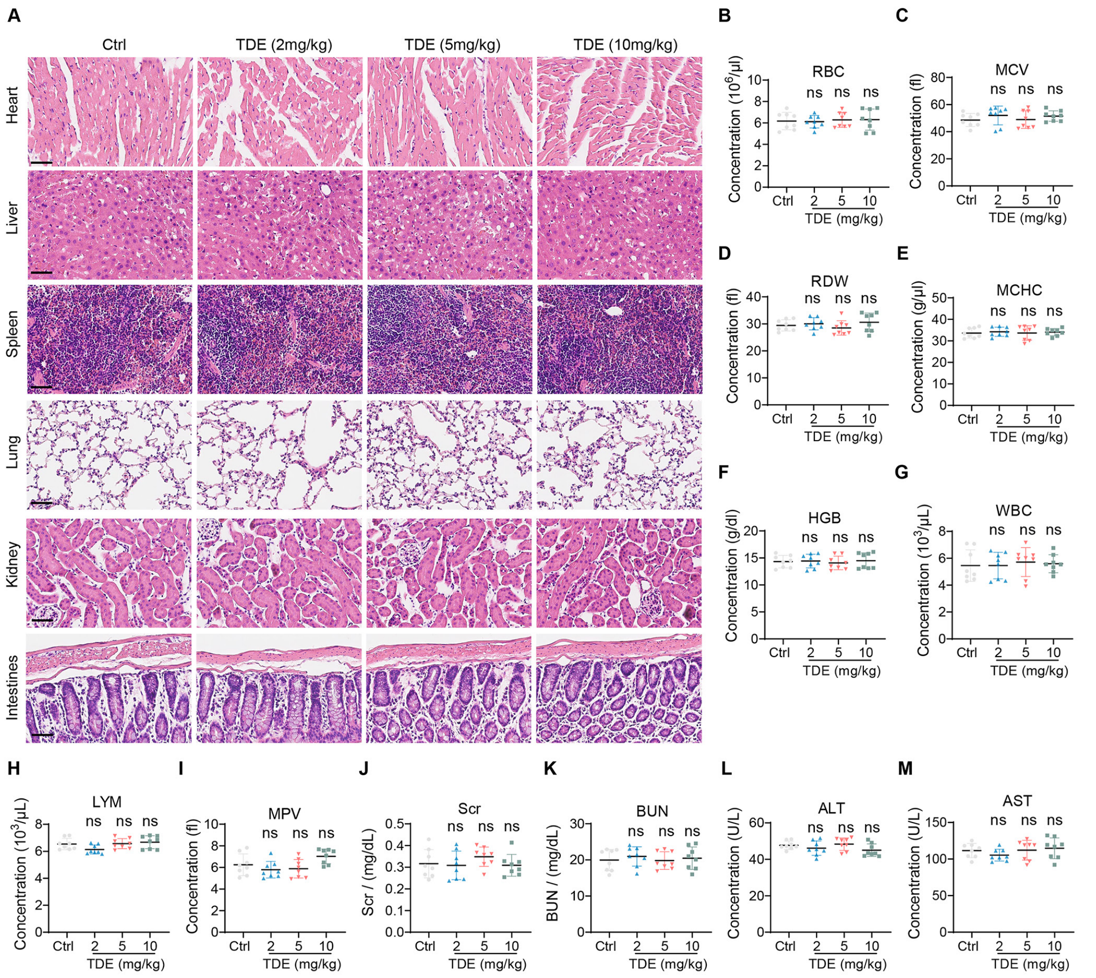

Supplementary Figure 8. *In vivo* Toxicity Assessment of TDE.

A C57BL/6J mice were intraperitoneally injected with different doses of TDE (control, 2, 5, 10mg/kg) for 28 days, and the major organs were removed for HE staining. Scale bar = 50 μm. **B–M** Blood samples were collected to perform hematological and hepatic/renal function tests in the mice (n = 8). RBC, red blood cell; MCV, mean corpuscular volume; RDW, red cell distribution width; MCHC, MCH concentration; HGB, hemoglobin; WBC, white blood cell; LYM, lymphocyte; MPV, mean platelet volume; Scr, creatinine; BUN, blood urea nitrogen; ALT, alanine transaminase; AST, aspartate transaminase. Data are shown as mean ± SD. ns: no significance.

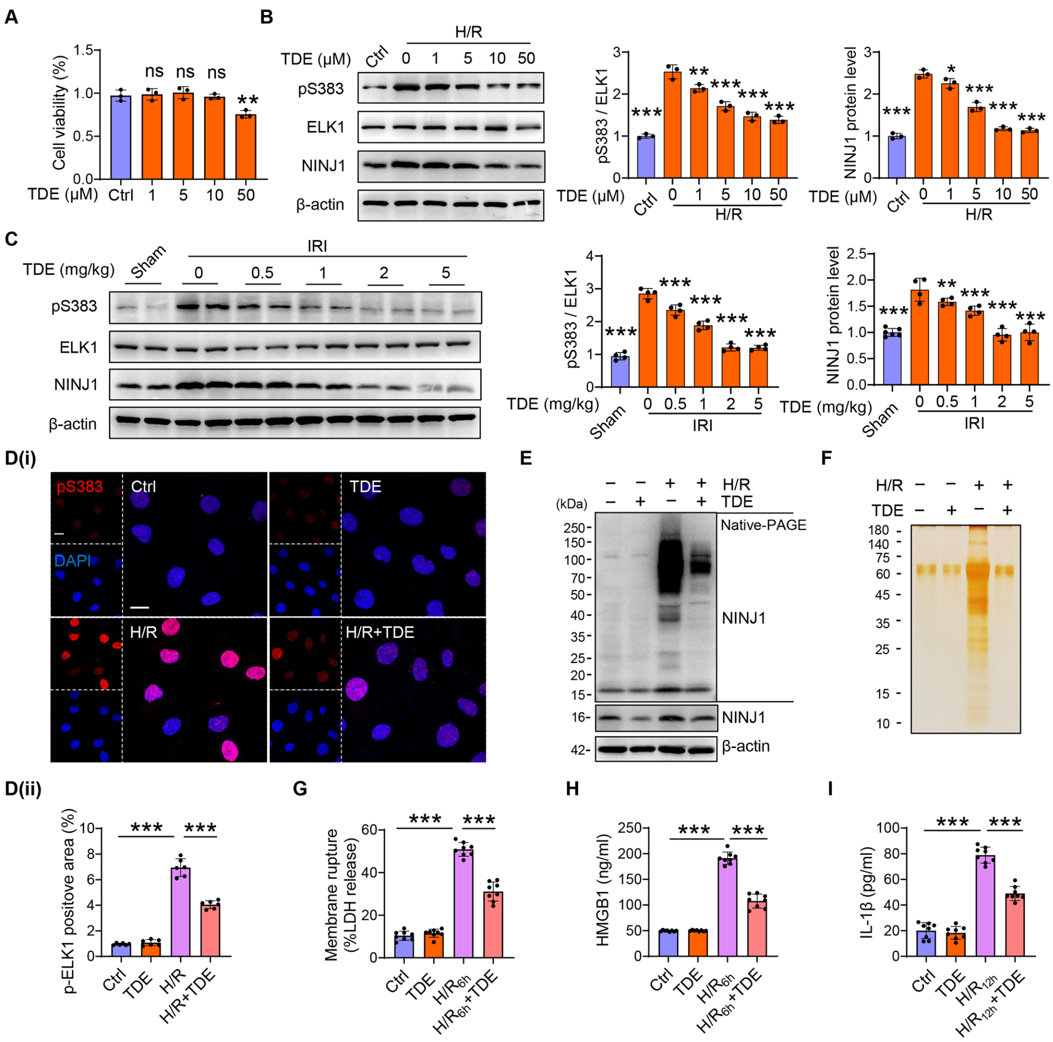

Supplementary Figure 9. Targeting ELK1 Ser^{383} phosphorylation by TDE treatment counteracts NINJ1-induced inflammation after AKI.

A The viability of HK-2 cells treated with control or multiple concentrations of TDE for 24 hours (n = 3). **B** Western blot analysis of p-ELK1 (S383), ELK1 and NINJ1 treated with different concentrations of TDE (n = 3). **C** C57BL/6J mice were intraperitoneally injected with different doses of TDE, and then kidneys were harvested for western blot analysis to evaluate the expression of p-ELK1 (S383), ELK1 and NINJ1 (n = 4). **D** Representative immunofluorescence staining and quantification of p-

ELK1 (S383) in HK-2 cells treated with TDE (10μM) under normoxia or H/R conditions (n = 6). Scale bar = 50 μm. **E** Native-PAGE analysis of endogenous NINJ1 in HK-2 cells following the described treatment in D. **F** Silver staining of released proteins in culture supernatant of HK-2 cells. **G-I** Release of LDH (G) and HMGB1 (H) in culture supernatant of HK-2 cells after 6 hours of reoxygenation and IL-1β (I) at 12 hours after reoxygenation (n = 8). Data are shown as mean ± SD. *$P < 0.05$; **$P < 0.01$; ***$P < 0.001$. ns: no significance.

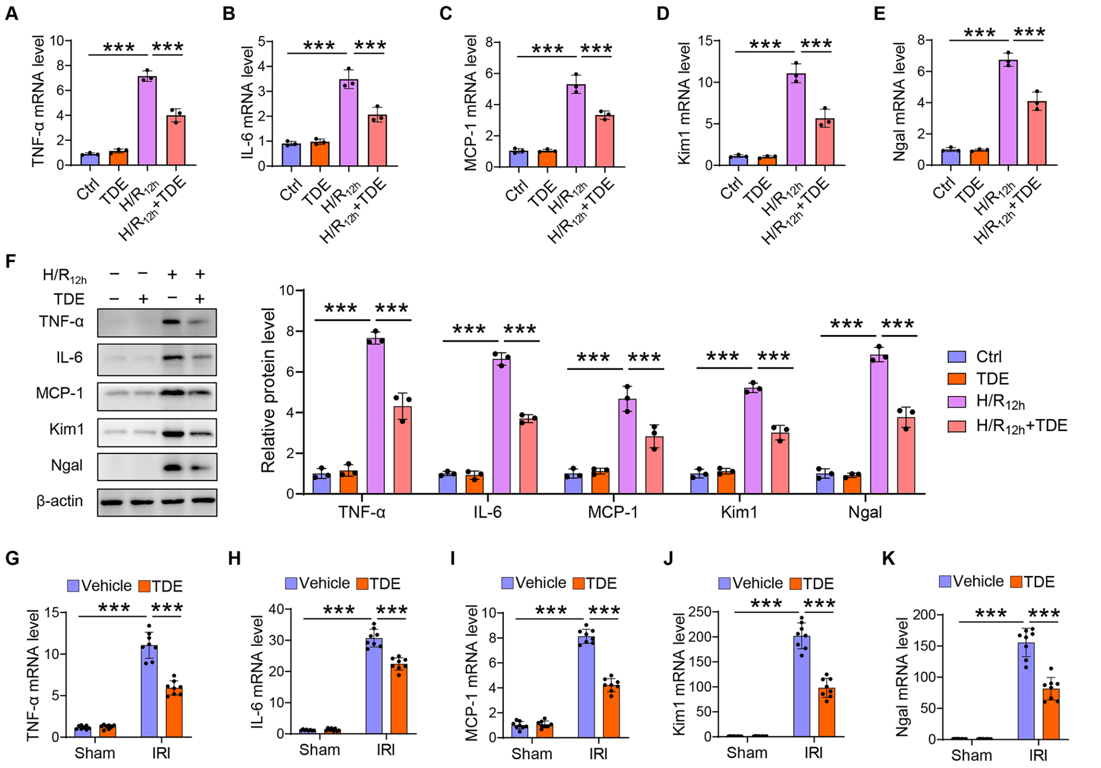

Supplementary Figure 10. Targeting ELK1 Ser383 phosphorylation by TDE treatment counteracts NINJ1-induced inflammation after H/R *in vitro* and IRI *in vivo*.

A-E qPCR analysis of TNF-α (A), IL-6 (B), MCP-1 (C), *Kim1* (D), and *Ngal* (E) expression in HK-2 cells treated with or without TDE (10μM) under normoxia or H/R conditions. (n = 3). **F** Western blot analysis of TNF-α, IL-6, MCP-1, *Kim1*, and *Ngal* in HK-2 cells following by 12 hours of reoxygenation (n = 3). **G-K** qPCR analysis of TNF-α (G), IL-6 (H), MCP-1 (I), *Kim1* (J), and *Ngal* (K) expression in mice injected with vehicle or TDE (2 mg/kg) before subjection to sham or IRI (n = 8). Data are shown as mean ± SD. ***$P < 0.001$.

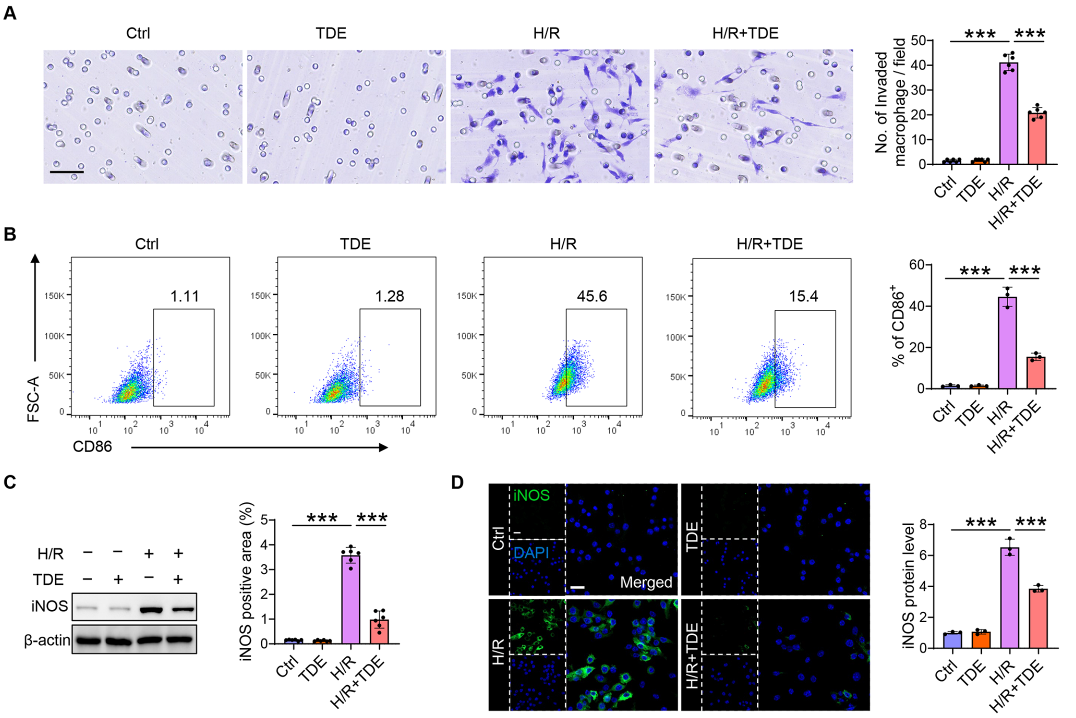

Supplementary Figure 11. TDE treatment counteracts Ninj1-mediate DAMP release in tubular epithelial cells induce the recruitment and activation of macrophages.

A Representative images and quantification of macrophages stained with crystal violet for the chemotaxis test (n = 6). Scale bar = 50 μm. **B** Representative flow cytometry (FC) analysis of the percentage of M1 macrophage in co-cultured with HK-2 cells for soluble factor communication study (n = 3). **C** Western blot analysis of iNOS expression in macrophages for soluble factor communication study (n = 3). **D** Representative immunofluorescence staining and quantification of M1 macrophage for soluble factor communication study (n = 3). Scale bar = 50 μm. Data are shown as mean ± SD. ***$P < 0.001$.

Supplementary Table

Supplementary Table 1. Clinical data of ATN and non-ATN patients examined.

Control subjects

Number	Age (year)	Sex	Scr (mg/dL)	BUN (mg/dL)
1	38	F	0.73077	8.276
2	33	F	0.72964	9.171
3	30	F	0.63462	10.401
4	37	F	0.78507	15.518
5	29	M	0.96493	12.694
6	32	F	0.68326	14.651
7	61	M	0.98643	16.273
8	35	F	0.71833	13.141
9	35	F	0.61991	10.317
10	19	M	0.67081	14.679
11	49	F	0.61086	16.552
12	38	F	0.65498	9.171
13	29	M	0.89480	16.217
14	20	F	0.74661	10.988
15	19	M	1.07692	15.993
16	52	F	0.67647	10.848
17	44	M	1.03054	15.154
18	52	F	0.74321	15.406
19	50	F	0.64819	13.589
20	63	F	0.65724	11.827

Subjects with acute tubular necrosis

Number	Age (year)	Sex	Scr (mg/dL)	BUN (mg/dL)
1	39	M	5.7805	38.137
2	39	F	8.0939	36.628
3	35	M	5.2251	52.201
4	63	M	2.1810	54.410
5	53	M	2.5373	48.343
6	37	M	1.7511	11.492
7	58	M	3.4231	45.044

8	64	F	5.7093	56.395
9	31	M	1.4593	16.776
10	34	M	1.5554	9.171
11	44	M	1.2240	22.759
12	56	F	1.3405	20.523
13	49	M	5.3812	68.390
14	45	M	7.3982	72.864
15	19	M	2.8054	102.026
16	39	F	3.0928	34.363
17	49	M	8.6210	55.920
18	31	F	4.3371	56.004
19	64	F	8.1188	55.053
20	59	M	4.7647	60.729
21	55	M	8.5735	53.711
22	42	M	10.1369	98.839
23	44	F	10.1844	90.982

Supplementary Table 2. The sequence sets for siRNA.

siRNA (human)	Primers
siNinj1	Forward: 5′- CUGGUGUUCAUCAUCGUGGUAdTdT<br>Reverse: 5′- UACCACGAUGAUGAACACCAGdTdT
siELK1	Forward: 5′- CCUGCUUCCUACGCAUACAUUdTdT<br>Reverse: 5′- AAUGUAUGCGUAGGAAGCAGGdTdT
siIRF1	Forward: 5′- CAGAUUAAUUCCAACCAAAdTdT<br>Reverse: 5′- UUUGGUUGGAAUUAAUCUGdTdT
siYY1	Forward: 5′- CGCUGAGUGUGGACCCUAAdTdT<br>Reverse: 5′- UUAGGGUCCACACUCAGCGdTdT
siNC	Forward: 5′- UUCUCCGAACGUGUCACGUdTdT<br>Reverse: 5′- ACGUGACACGUUCGGAGAAdTdT

Supplementary Table 3. Primary Antibody list.

**Antibodies**	**Source**	**Identifier**
Anti-Ninj1	BD Transduction Laboratories™	610777
Anti-Ninj1	GeneTex	GTX31596

Anti-Ninj1	R&D Systems	MAB5105
Anti-ELK1	Proteintech Group	27420-1-AP
Anti-p-ELK1 (Ser383)	Thermo Fisher Scientific	PA5-104832
Anti-p-ELK1	Santa Cruz Biotechnology	sc-8406
Anti-p-ELK1 (Ser389)	Thermo Fisher Scientific	PA5-104833
Anti-p-ELK1 (Thr417)	Thermo Fisher Scientific	PA5-36642
Anti-F4/80	Proteintech Group	28463-1-AP
Anti-Ly6G	Santa Cruz Biotechnology	sc-53515
Anti-β-Actin	abclonal	AC004
Anti-α-SMA	abcam	Ab7817
Anti-Fibronectin	abcam	AB2413
Anti-TNF-α	AiFang biological	AFRM9306
Anti-IL6	Affinity Biosciences	DF6087
Anti-MCP-1	HUABIO	HA500267
Anti-Kim1	Santa Cruz Biotechnology	sc-518008
Anti-Ngal	Santa Cruz Biotechnology	sc-515876
Anti-iNOS	HUABIO	ER1706-89
CD86 eFluor 647	Invitrogen	51-0869-42
F4/80 FITC	Invitrogen	11-4801-85

Supplementary Table 4. The primer sets for human.

Gene (human)	Primer Sequence (5'-3')	Product length
Ninj1	Forward: TCAAGTACGACCTTAACAACCCG<br>Reverse: TGAAGATGTTGACTACCACGATG	102 bp
ELK1	Forward: TCCCTGCTTCCTACGCATACA<br>Reverse: GCTGCCACTGGATGGAAACT	144 bp
β-actin	Forward: CATGTACGTTGCTATCCAGGC<br>Reverse: CTCCTTAATGTCACGCACGAT	250 bp
Kim1	Forward: TGTCTGGACCAATGGAACCC<br>Reverse: GGCAACAATATACGCCACTGT	134 bp
Ngal	Forward: TCACCCTCTACGGGAGAACC<br>Reverse: GGTCGATTGGGACAGGGAAG	117 bp
TNF-α	Forward: TGCACTTTGGAGTGATCGGC<br>Reverse: CTCAGCTTGAGGGTTTGCTAC	146 bp

MCP-1	Forward: CAGCCAGATGCAATCAATGCC<br>Reverse: TGGAATCCTGAACCCACTTCT	190 bp
IL6	Forward: ACTCACCTCTTCAGAACGAATTG<br>Reverse: CCATCTTTGGAAGGTTCAGGTTG	149 bp
IRF1	Forward: ATGCCCATCACTCGGATGC<br>Reverse: CCCTGCTTTGTATCGGCCTG	204 bp
YY1	Forward: AGCCCTTTCAGTGCACGTT<br>Reverse: GTCTCCGGTATGGATTCGCA	89 bp

Supplementary Table 5. The primer sets for mouse.

Gene (mouse)	Primer Sequence (5'-3')	Product length
Ninj1	Forward: GAGTCGGGCACTGAGGAGTAT<br>Reverse: CGCTCTTCTTGTTGGCATAATGG	136 bp
ELK1	Forward: TTGTGTCCTACCCAGAGGTTG<br>Reverse: GCTATGGCCGAGGTTACAGA	95 bp
β-actin	Forward: TGTTACCAACTGGGACGACA<br>Reverse: GGGGTGTTGAAGGTCTCAAA	165 bp
Kim1	Forward: AGCAGTCGGTACAACTTAAAGG<br>Reverse: ACTCGACAACAATACAGACCAC	101bp
Ngal	Forward: GGAGCGATCAGTTCCGGG<br>Reverse: CTGATCCAGTAGCGACAGCC	181 bp
TNF-α	Forward: CCTGTAGCCCACGTCGTAG<br>Reverse: GGGAGTAGACAAGGTACAACCC	148 bp
MCP-1	Forward: TAAAAACCTGGATCGGAACCAAA<br>Reverse: GCATTAGCTTCAGATTTACGGGT	120 bp
IL6	Forward: CTGCAAGAGACTTCCATCCAG<br>Reverse: AGTGGTATAGACAGGTCTGTTGG	131 bp

Supplementary Table 6. Putative binding sequences of ELK1 in Ninj1 promoter region.

Name	Start	End	Predicted sequence
ELK1	-1638	-1629	AACCCGGGAG
ELK1	-1498	-1489	ACAAAGGAAA

ELK1	-908	-899	CTTCTGGAAA
ELK1	-885	-876	CTGCCGCAAG
ELK1	-560	-551	AGGCAGGAAA

Supplementary Table 7. The primer sets for ChIP.

Gene (human)	Primer Sequence (5'-3')	Product length
Ninj1	Forward: ATGCCCACTCACTCCTACC<br>Reverse: TGGCCACTCTATTTCCAGA	104 bp
